# Cardiovascular risk of veterans’ football: An observational cohort study with follow-up

**DOI:** 10.1371/journal.pone.0297951

**Published:** 2024-04-05

**Authors:** Florian Egger, Tilman Schilling, Sybille Baumann, Tim Meyer, Jürgen Scharhag

**Affiliations:** 1 Institute of Sports and Preventive Medicine, Saarland University, Saarbrücken, Germany; 2 Department of Sport and Human Movement Science, Centre for Sport Science and University Sports, Sports Medicine, Exercise Physiology and Prevention, University of Vienna, Wien, Austria; University of Dundee, UNITED KINGDOM

## Abstract

**Background:**

The cardiac stress for veteran football players during match is considerable. In this specific elderly population, the kinetics of exercise-induced cardiac troponin I (cTnI) and B-Type natriuretic peptide (BNP) could potentially be related to cardiovascular risk factors (CVRF) and cardiovascular disease and are therefore be investigated for their usefulness as an complement to established screening measures.

**Methods:**

cTnI and BNP was measured in 112 veteran football players (age: 51 ± 10 years) within 30 minutes pre- and post-match. Players with elevated cTnI (cTnI-positive) and a control group (out of the 112 veteran players) with normal cTnI (cTnI-negative) underwent cardiac follow-up 4.2 ± 3.5 months post-match, comprising history, resting and stress ECG (including 30 minutes pre- and post cTnI and BNP), and echocardiography.

**Results:**

In 33 players (29%) cTnI and in 6 players BNP (5%) exceeded the upper range limit for increased risk of myocardial damage (cTnI ≥ 5 ng/l) and myocardial wall stress (BNP ≥ 100 pg/ml) post-match, respectively. No correlation was observed between Δ cTnI (pre- vs. post-match) and the number of CVRF (r = -0.06, p = 0.50). Follow-up was conducted in 62 players (31 cTnI-positive and 31 cTnI-negative players) of which 6 (10%, 3 cTnI positive and 3 cTnI negative players) had cardiac abnormalities (hypertrophic cardiomyopathy n = 2, coronary artery disease n = 2, coronary artery anomaly n = 1, hypertensive heart disease n = 1).

**Conclusion:**

Veterans’ football matches elicit increases in BNP and particularly cTnI in a considerable number of players. However, these biochemical alterations do not indicate acute cardiac damage as evidenced by follow-up. Routine determination of cardiac biomarkers is unlikely to improve cardiovascular screening in veteran football players.

## Introduction

Recreational football has shown to have beneficial effects on the cardiovascular risk profile in untrained individuals and veteran football players [1–3]. However, beyond the age of 35 years, the incidence of sports-related sudden cardiac death increases, primarily due to coronary artery disease (CAD) [4–6]. In veterans’ football, the cardiac and metabolic stress in both training and competition is considerable, potentially exposing players with underlying cardiovascular diseases to some risk [7]. In contrast to the majority of older people exercising at low to moderate intensity, veteran football players represent a specific population of older people who are intermittently exposed to high intensity exercise [7].

Post-exercise elevated biomarkers, such as cardiac troponin (cTn) and B-type natriuretic peptide (BNP), may indicate both a physiological response to exercise or myocardial damage and increased myocardial wall stress [8, 9]. Different from cardiac emergencies, exercise-induced cTn kinetics show their peak (within several hours) and recovery (within 2–3 days) earlier than in myocardial necrosis [8, 10, 11]. While previous literature focused mainly on cTn elevations after prolonged exercise [10–15], few studies exist on cTn response in team sports with high cardiac stress [16–18]. In football, post-match cTn has been studied predominantly in children or adolescents and, so far, only in a small group of adults [19–21]. Given the high number of veteran football players worldwide, it seems appropriate to assess the cardiac health status in this potentially vulnerable middle-aged population using cardiac biomarkers established in routine clinical practice. In this setting a close follow-up seems to be a sufficient approach in asymptomatic individuals without clinical abnormalities [8]. The aim of this study is to investigate the cardiovascular risk of veteran football players during match, measured by cTn and BNP and further assessed by follow-up, in order to derive recommendations for the players’ screening program.

## Materials and methods

This prospective study was conducted in the German federal state of Saarland. The study was carried out in accordance with the declaration of Helsinki and approved by the local ethics committee (Ärztekammer des Saarlandes, Saarbrücken, Germany; approval number: 81/15).

### Subjects

Subjects were recruited by contacting all veterans football clubs registered with the local football association. Only subjects regularly participating in veterans’ football matches were included. Exclusion criteria were acute infections, severe internal diseases with limited exercise capacity, or orthopedic injuries and pain-related musculoskeletal limitations. All subjects were fully informed about the experimental procedures and gave written informed consent prior to participation. All relevant data are presented in tabular form (S1 Data).

### General design

**Fig 1:**
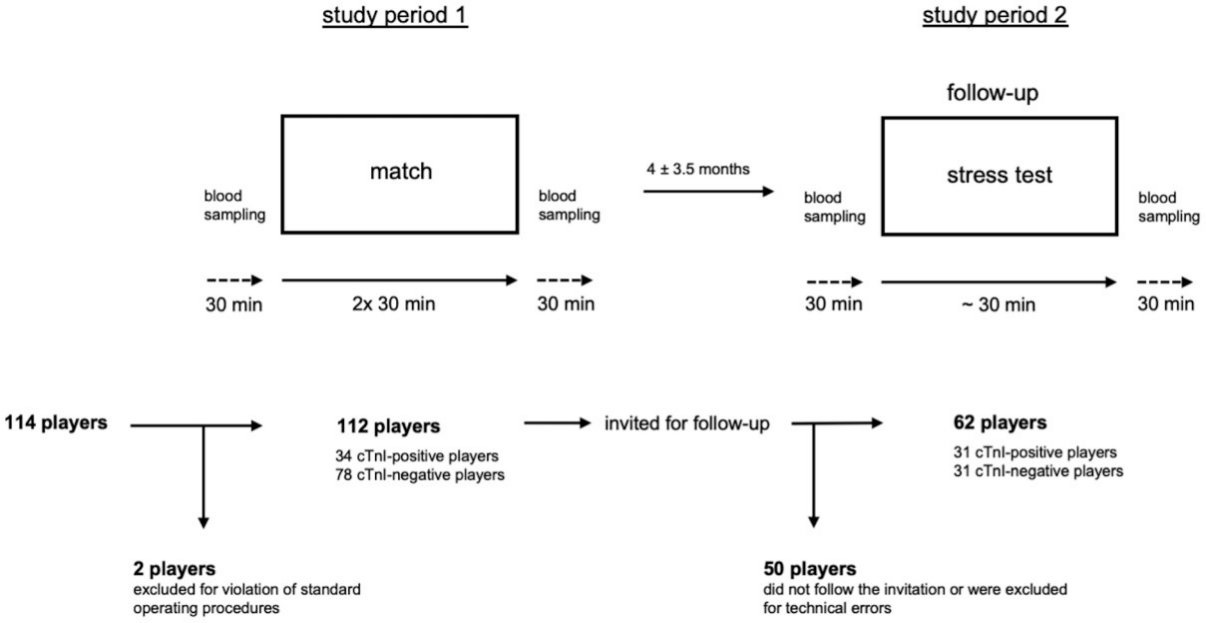
Schematic representation of the general design. After recruitment of 114 veteran football players 2 players were excluded for violation of standard operating procedures. A total of 112 players participated in a regular league match (2 halves of 30 minutes each) and underwent blood sampling for cardiac biomarkers (cTnI and BNP) within 30 minutes before and after the match. Blood analysis revealed 34 cTnI-positive and 78 cTnI-negative players. Subsequently, all subjects (n = 112) were invited for cardiac follow-up. Of them, 50 players did not follow the invitation or were excluded for technical errors. Within a period of 4 ± 3.5 months 62 players (31 cTnI-positive players and 31 cTnI-negative players) completed cardiac follow-up including a stress test in which blood samples (cTnI and BNP) were collected again 30 minutes before and after the test.

The study consisted of two phases: The first one involved the assessment of cardiac stress during a veterans’ football match by measuring cardiac troponin I (cTnI) and BNP pre- and post-exercise. The second one comprised a follow-up of players with elevated pre- or post-match cTnI, defined as cTnI-positive players, and age-matched controls (out of total study population) with normal pre- and post-match cTnI values, defined as cTnI-negative players. An overview of the design, including a flowchart of subject recruitment, is provided in Fig 1.

### Cardiac stress during football (study period 1)

Cardiac stress of players was investigated in 6 veterans’ football league matches over a 9-month period. Each match consisted of two 30-minute halves and a 15-minute break. A team consisted of 11 players who could be substituted as often as desired. Individual playing time was not registered. Prior to the match medical assessment took place in the club house of the home teams. All players completed a standardized health survey concerning their current symptoms, pre-existing diseases and medication intake (Physical Activity Health Questionnaire, PAR-Q) [22]. Subsequently, a comprehensive medical history was taken by a physician. After the match the same physician conducted a second history via telephone interview to clarify any ambiguities and to obtain more detailed medical information (which was not always possible in the pre-match setting). Within 30 minutes before the match a venous blood sample for screening purposes was taken to determine a complete blood count (Coulter AcT 5 diff autoloader [AL] hematology analyzer, Beckman Coulter, Fullerton, USA), creatine kinase, urea, creatinine (UniCel DxC 600 Synchron Clinical System, Beckman Coulter, Fullerton, USA). The same blood sample was analyzed for high-sensitivity cTnI and BNP (Access AccuTnI+3 Troponin I Assay and Abott Triage BNP both for use on the Access 2 Immunoassay System, Beckman Coulter, Fullerton, USA). Positive cTnI was defined as values above assay specific upper range limit (URL) 99^th^ percentile ≥ 5 ng/L. A follow-up blood sample with the same parameters was taken 30 minutes after the match. Serum blood samples were always centrifuged on site within 10 minutes after collection (mobile centrifuge universal 320, Hettich, Tuttlingen, Germany) and stored together with the plasma samples in cool boxes for a maximum of 2 hours before being transported to the laboratory where they were frozen at minus 18°C. When sampling was completed, all samples were thawed at the same time to avoid fluctuations due to different analysis times. A cTnI concentration of less than 5 ng/L was established as the cut-off value for low vs. increased probability of myocardial damage with a negative predictive value of >99.4%, as previously reported [23, 24]. Likewise a BNP cut-off concentration of < 100 pg/ml was used to exclude increased myocardial wall stress [25, 26].

### Follow-up (study period 2)

Within a period of 4.2 ± 3.5 months post-match, cTnI-positive players and cTnI-negative players (serving as an age-matched control group) as well as players with elevated BNP were invited for a comprehensive cardiac follow-up. A series of routine examinations was performed by a cardiologist, including history, physical examination, resting and stress ECG (incremental cycle test with 50 W steps every 3 min starting from 50 W or 100 W until volitional exhaustion on an electrically braked cycle ergometer [Excalibur Sport, Lode, Groningen, The Netherlands]), and echocardiography. Before and 30 minutes after the stress test cTnI and BNP values were determined for both groups.

### Outcome measures

The changes in cardiac biomarkers cTnI and BNP pre- and post-exercise and the percentage of cardiovascular disease in veteran soccer players were the primary outcome measures of this study. The number of cardiovascular risk factors (CVRF) and their association with cardiac biomarkers were selected as secondary outcome measures.

### Statistics

Since there were very limited data published to establish baseline assumptions, no formal sample size estimation was performed. When normal distribution was confirmed by a Shapiro-Wilk test, data were reported as mean ± standard deviation. Non-normally distributed data were expressed as median and interquartile range (IQR). Differences for independent variables were tested by the unpaired Student t-test. Associations between two variables were calculated using the Pearson correlation coefficient and, as the non-parametric equivalent, the Spearman rank correlation. A value of p < 0.05 for the α-error was considered statistically significant. Statistical analyses were performed using GraphPad (version 9.0).

## Results

The baseline characteristics of 112 players included in this study are presented in Table 1.

**Table 1:** Baseline characteristics of 112 veteran football players*.

Age [years]	51 ± 10
Height [cm]	176 ± 7
Weight [kg]	83 ± 11
BMI [kg/m^2^]	26.5 ± 2.7
**Cardiovascular risk factors, n (%)**	
Type 2 diabetes,	9 (8)
Hypertension	17 (15)
Hypercholesterolemia	20 (18)
Smoking	50 (45)
Obesity	12 (11)
Family history	18 (16)
**Number of cardiovascular risk factors, n (%)**	
0	38 (34)
1	44 (39)
2	14 (13)
3	10 (9)
4	6 (5)
**Known cardiovascular diseases, n (%)**	
Coronary artery disease	7 (7)
Myocarditis in the past	3 (3)
Arrhythmia	3 (3)
Hypertrophic obstructive cardiomyopathy*	1 (1)
Aortic stenosis	1 (1)
Carotid artery stenosis	1 (1)
**Medication, n (%)**	
Aspirin or clopidogrel	6 (5)
Oral anticoagulant	1 (1)
ACE-inhibitor	9 (8)
Renin-angiotensin inhibitor	3 (3)
Beta-blocker	3 (3)
Diuretics	1 (1)
Statins	10 (9)
Metformin	4 (4)
Antiarrhythmics	1 (1)
L-Thyroxin	5 (5)
**Blood parameters**	
Erythrocyte count [million/mm^3^]	4.8 ± 0.3
Hemoglobin [g/dl]	14.8 ± 0.9
Hematocrit [%]	44.3 ± 2.4
White-cell count [thousand/mm^3^]	7.0 (5.8–8.0)
Platelet count [thousand/mm^3^]	254.6 ± 51.7
Creatine kinase [U/L]	154 (108–237)
Urea [mg/dl]	37.2 ± 7.2
Creatinine [mg/dl]	0.98 ± 0.14
**Cardiac biomarkers**	
Troponin I [ng/L]	0 (0–1)
BNP [pg/ml]	27 (21–41)

Plus-minus values are means ± SD. Alternatively, in cases of skewed distributions, data are presented as medians and interquartile range.

*treated by myectomy

### Pre- and post-match concentrations of cTnI and BNP

None of the players reported cardiac symptoms before, during, or after the match.

In 33 of 112 players (29%) cTnI was elevated after the match and in 13 players of them also before the match. In 5 players (4%) cTnI was elevated pre-match but not post-match. In total, there were 38 cTnI-positive players (34%). In 29 cTnI-positive players cTnI increased [Δ cTnI 11 (9–18) ng/l] and in 9 cTnI-positive players [Δ cTnI 8 (6–18) ng/l] decreased from pre- to post-match.

**Fig 2:**
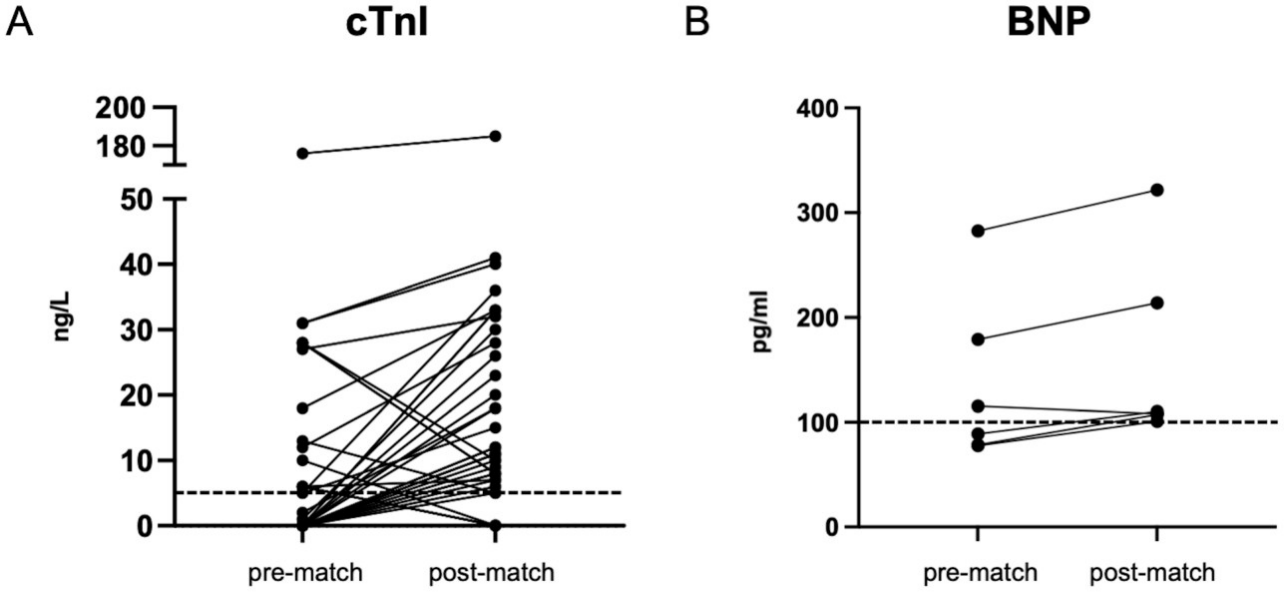
Individual pre- and post-match elevations of cTnI (A, n = 38) and BNP (B, n = 6). The dashed line indicates the cut-off value for cTnI < 5 ng/L and for BNP < 100 pg/ml.

In 6 of 112 players (5%) BNP was elevated after the match and in 3 players (3%) before the match. BNP increased in 5 players (Δ BNP 29 ± 8 pg/ml) and decreased in 1 player (115 to 108 pg/ml) from pre- to post-match. Individual pre- and post-match elevations of cTnI and BNP above the respective cut-off values are illustrated in Fig 2.

No correlation was observed between Δ cTnI (pre- vs. post-match) and the number of CVRF (r = -0.06, p = 0.50). Post-match cTnI did not correlate with post-match BNP (r = -0.01; p = 0.97), creatine kinase (r = -0.11; p = 0.24), urea (r = -0.01; p = 0.89) and creatinine (r = - 0.02; p = 0.84). Likewise post-match BNP did not correlate with post-match creatine kinase (r = -0.10; p = 0.27), urea (r = 0.07; p = 0.47) and creatinine (r = 0.06; p = 0.55). The association of individual cTnI and BNP elevations with CVRF and other internal diseases from a total of 42 players is presented in Table 2.

**Table 2:** Individual association of elevated cTnI and BNP with history-based cardiovascular risk factors and other internal diseases in 42 veteran football players.

Player No.	cTnI pre [ng/l]	cTnI post [ng/l]	BNP pre [pg/ml]	BNP post [pg/ml]	No. of CVRF	CVRF	Other internal diseases
1	**31**	**41**	20	23	1	SM (10 py)	Bronchial asthma
2	**6**	1	38	53	1	SM (30 py)	Bronchial asthma
3	**28**	**8**	41	42	2	HTN, SM (3 py)	-
4	0	**10**	20	24	4	HCL, HTN, OB, SM (13 py)	-
5	**5**	**36**	61	47	4	HCL, HTN, OB, SM (15 py)	**CAD with history of MI**
6	**10**	0	17	22	3	FH, HTN, SM (20 py)	-
7	0	0	**78**	**101**	4	T2D, HTN, HCL, SM (15 py)	**Aortic stenosis**
8	0	**11**	52	45	0	-	Crohn’s disease, Parkinson’s disease
9	0	0	79	**107**	1	HTN	Prostate cancer
10	0	**26**	25	28	2	HTN, SM (13 py)	-
11	**27**	**32**	33	38	0	-	-
12	0	0	**282**	**322**	2	HTN, HCL	**CAD with history of MI**
13	0	**12**	42	47	1	FH	-
14	0	**5**	15	17	0	-	Bronchial asthma
15	0	**33**	32	42	0	-	-
16	1	**30**	**179**	**214**	1	SM (25 py)	**History of myocarditis**
17	0	**11**	17	45	2	SM (10 py), OB	-
18	0	**9**	34	43	0	-	-
19	**18**	**33**	52	50	3	FH, HCL, SM (15 py)	**HOCM**
20	0	**23**	16	23	2	FH, HCL	-
21	**12**	**28**	25	25	0	-	-
22	0	**7**	34	37	2	FH, SM (20 py)	Hypothyroidism
23	**5**	**15**	21	26	3	FH, SM (20 py)	-
24	1	0	89	**110**	1	SM (15 py)	-
25	2	18	22	31	1	SM (25 py)	-
26	0	**12**	41	39	0	-	-
27	0	**33**	25	30	1	SM (20 py)	-
28	0	**8**	31	30	1	FH	**History of myocarditis**
29	**13**	**5**	32	42	2	T2D, SM (45 py)	-
30	**28**	**8**	22	34	1	OB	-
31	**28**	**10**	14	19	3	FH, HCL, SM (5 py)	
32	**31**	**40**	21	34	0	-	-
33	**6**	0	28	21	4	HCL, HTN, SM (1 py), OB	-
34	0	**20**	**115**	**108**	1	SM (5 py)	-
35	**6**	**7**	18	26	0	-	-
36	0	**6**	50	39	0	-	-
37	0	**8**	18	22	0	-	-
38	0	**11**	27	25	0	-	-
39	0	**18**	36	40	1	FH	-
40	**176**	**185**	35	33	2	FH, HCL	**Suspected paroxysmal tachycardia**
41	**6**	0	47	48	0	-	-
42	**6**	0	49	63	1	HTN	**History of myocarditis**

Pre, pre-match; post, post-match; cTnI, cardiac troponin I; BNP, B-type natriuretic peptide; CVRF, cardiovascular risk factors; FH, family history; HCL, hypercholesterolemia; HTN, hypertension; SM, smoking (current and former smokers combined); py, pack years; T2D, type 2 diabetes; OB, obesity; bold type, cardiovascular diseases.

Based on a comprehensive history, cardiovascular diseases (Table 1) were identified in 16 players, of whom 8 players (50%) had elevated cTnI or BNP levels and 5 players (31%) elevated post-match cTnI levels (Table 2). The player with the highest post-match BNP (322 pg/ml) had a history of coronary bypass surgery and the player with the highest cTnI (185 ng/l) was under investigation for paroxysmal tachycardia. Median and IQR of elevated post-match cTnI and BNP were 12 (8–30) ng/l and 109 (108–188) pg/ml, respectively.

### Cardiac follow-up (study period 2)

From 112 players cardiac follow-up was attended by 31 cTnI-positive players (including 1 player with elevated BNP) and 31 age-matched cTnI-negative players (control group) 4.2 ± 3.5 months (range: 2.9–15.0 months) post-match. Since match day none of them had cardiovascular symptoms or experienced a major adverse cardiovascular event (myocardial infarction or stroke). In 6 of the 62 players (10%) cardiac abnormalities were detected by history (1 cTnI-positive and 2 cTnI-negative players, 5%) or first diagnosed (1 cTnI-negative player and 2 cTnI-positive palyers, 5%) during cardiac follow-up. Anthropometric data, distribution of CVRF, relevant cardiac diseases, and medication use of all players undergoing cardiac follow-up are provided separately (S1 Table).

#### Echocardiography

In all players undergoing cardiac follow-up left ventricular systolic function was preserved (ejection fraction > 52%) without significant wall motion abnormalities (only one player showed asynchronous motion of the intraventricular septum in the presence of left bundle branch block) [27]. Diastolic function measured as the ratio of transmitral peak early (E wave) to late (A wave) diastolic filling velocity was reduced in 24 of 62 players (39%; E/A 0,7 ± 0,1). In one cTnI-positive player echocardiography revealed a previously unknown hypertrophic cardiomyopathy (HCM) with an inferoseptal wall thickness of 33 mm and intraseptal hyperechogenic regions. A second cTnI-positive player had a history of hypertrophic obstructive cardiomyopathy (HOCM) treated by myectomy years ago showing a normal pressure gradient across the left ventricular outflow tract and normal systolic function at the time of examination. A third cTnI-positive player showed an atypically dilated origin of the right coronary artery. Further evaluation by computed tomography angiography revealed an anomalous origin of the left circumflex artery from the right coronary artery. A cTnI-negative player with hypertension showed mild concentric hypertrophy (intraventricular septum and posterior wall 13 mm each) as a feature of hypertensive heart disease (HHD) with diastolic dysfunction (E/A ratio 0.6, e`_lateral_ 7 cm/s). Two cTnI-negative players with history of stable CAD (including one with a muscle bridge) showed preserved left ventricular ejection fraction without regional wall motion abnormalities.

#### ECG and stress test

Resting ECGs were abnormal in 3 of 62 players (5%). The cTnI-positive player with HCM and the cTnI-negative player with HHD both showed T-wave inversions in the precordial leads. A cTnI-negative player without significant abnormalities in echocardiography showed left bundle branch block (QRS 173 ms). During stress test, none of the players showed signs of myocardial ischemia (significant ST-segment or T-wave changes) or clinically relevant arrhythmias. Isolated monomorphic premature ventricular beats were observed in 6 cTnI-positive and 6 cTnI-negative players, respectively. Maximum cycling performance [210 ± 46 W (2.5 ± 0.5 W kg^-1^) and 218 ± 36 W (2.7 ± 0.4 W kg^-1^)] and maximum heart rate (166 ± 16 bpm and 167 ± 13 bpm) did not differ (p = 0.44) between cTnI-positive and cTnI-negative players, respectively. None of the players reported cardiac symptoms.

#### Cardiac biomarkers

Before the stress test 15 of 31 cTnI-positive players (48%) and 13 of 31 cTnI-negative players (42%) showed cTnI elevations. After the stress test, cTnI was elevated in 26 of 31 cTnI-positive players (84%) and in 9 of 31 cTnI-negative players (29%). In 32 of these 35 players (91%) with elevated post-exercise cTnI, stress test or echocardiography revealed no abnormalities.

**Fig 3:**
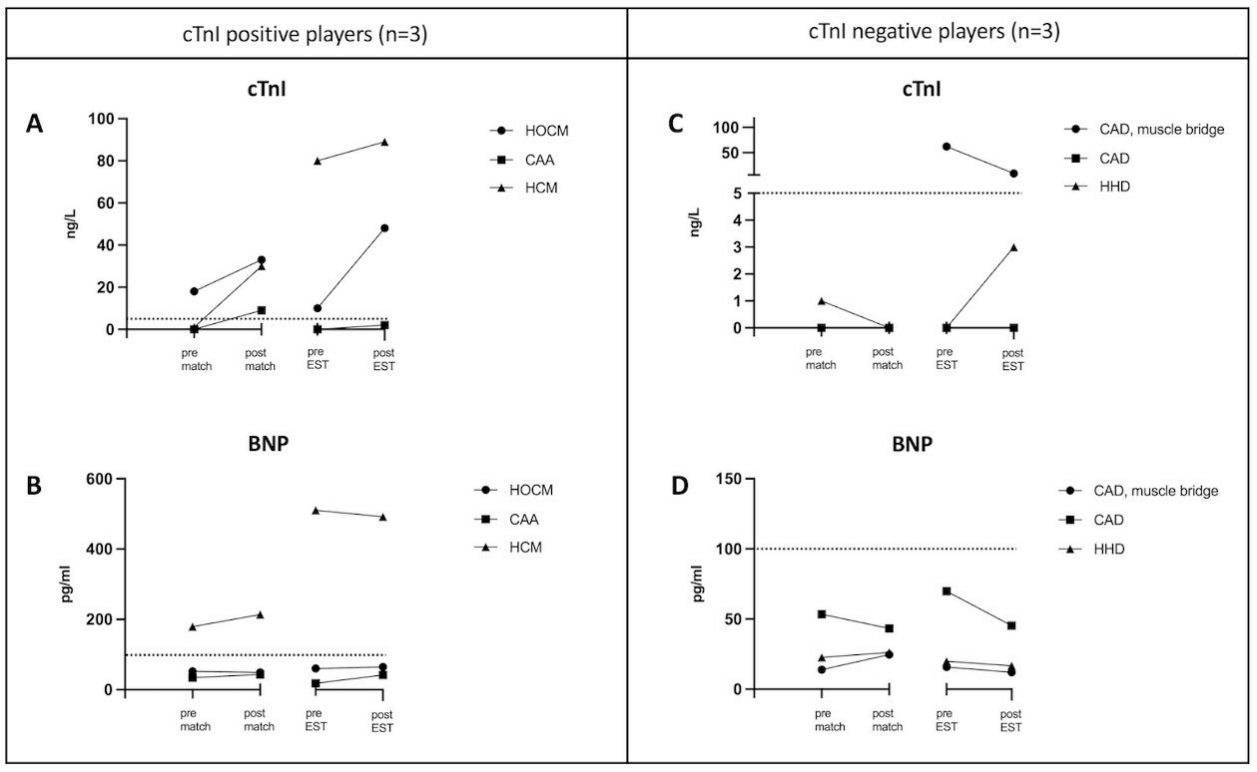
Individual cTnI and BNP concentrations from the 6 players with cardiac abnormalities during cardiac follow-up. The dashed line indicates the cut-off value for cTnI < 5 ng/L and for BNP < 100 pg/ml. HOCM, hypertrophic obstructive cardiomyopathy; CAA, coronary artery anomaly; HCM, hypertrophic cardiomyopathy; CAD, coronay artery disease; HHD, hypertensive heart disease.

The player diagnosed with HCM by echocardiography showed elevated BNP before and after stress test. All other players had normal BNP values at cardiac follow-up. After the stress test the 3 players with HCM, HOCM, and CAD with muscle bridge showed elevated cTnI and the 3 players with CAA, CAD, and HHD had normal cTnI. The individual cTnI and BNP concentrations from the 6 players with cardiac abnormalities are shown in Fig 3.

## Discussion

This was the first study to investigate the cardiovascular risk of veterans’ football by assessing the match-induced acute response of cardiac biomarkers in conjunction with a cardiac follow-up. It has been shown that a conventional veterans’ football match elicits post-match cTnI and BNP elevations above the respective cut-off values for cardiac stress and myocardial wall stress in 29% and 5% of players, respectively. Changes in cTnI did not correlate with CVRF. Given that none of the players reported cardiac symptoms during or after the match or experienced a cardiovascular event in the time to follow-up, veterans’ football does not appear to place players at particular cardiovascular risk.

### Clinical significance of elevated cardiac biomarkers after exercise

In this study, players identified with cardiovascular diseases by history showed elevated post-exercise cTnI or BNP only in half of the cases. At follow-up, post-exercise cTnI or BNP were elevated in players with HCM, HOCM, and CAD with muscle bridge, but remained negative in players with HHD, CAA and CAD. Taken together, the clinical significance of elevated cardiac biomarkers in veteran football players after match (non-superiority to medical history) or stress test remains questionable. However, in 31% and 50% of veteran football players with cTnI increases after match and stress test, respectively, an underlying cardiovascular disease was present. This observation is much less pronounced in younger athletes [28, 29]. Of 21 asymptomatic cTnT-positive athletes, all but one (critical CAD) showed no signs of myocardial damage 3 months after an endurance competition during a comprehensive cardiac assessment [29]. Furthermore, acute post-race elevation of cTnT in 5 marathon runners was unrelated to left-ventricular function assessed within 1 hour after competition [28].

In 38 marathon runners right ventricular dysfunction assessed 20 minutes post-race correlated with cTnT increase [30], which was a transient phenomenon also observed in 14 marathon runners examined immediately after competition [31]. In contrast, a recent study reported that in older long-distance walkers elevated cTnI after 30 to 55 km of walking was a predictor of future mortality and cardiovascular events after a mean follow-up of 43 months [13]. Nevertheless, cardiovascular morbidity was estimated based on self-reports, and post-exercise cTnI prevalence was low, which is not transferable to higher volume, higher intensity sports [13].

### Response of cardiac biomarkers to football

Previous studies have investigated the response of cTn and BNP to football in young and healthy individuals. The results are inconsistent and can most likely be explained by differences in timing of blood samples, playing level, training intensity and duration, troponin subunits measured (cTnI or cTnT), gender, and a wide age range [18–20, 32]. Only modest increases of BNP and cTnI were observed during recovery (5 min, 1h, 3h, 6h, 12h, 24h post) after an indoor football match in young healthy (11 males, 10 females) football players (only 2 females exceeded the URL for BNP and 2 males the URL for cTnI at all sampling points) [32]. Notably, pre- and post-match cTnI peak values of the male football players were 218 ng/L and 237 ng/L, respectively [32], which were both higher than the peak post-match cTnI (185 ng/l) in a veteran football player with suspected paroxysmal tachycardia in the present study [32]. However, the two highest post-match BNP values (322 pg/ml, 214 pg/ml) of veteran football players with CAD and history of myocarditis (Table 2) were higher than post-match peak BNP (108 pg/ml) in healthy male football players [32]. There is also evidence that cTn remains normal in response to football. In 10 male adult football players, cTnT was not detected in any blood sample before, immediately after, and 24 hours after a competitive match [18]. Furthermore, it has been shown that the magnitude of post-match cTnT increase did not differ between healthy young adults and children [21]. Given the inconsistent findings even in young (adult) football players regarding acute post-match response to cardiac biomarkers, comparability to veteran football players with previous cardiovascular diseases is limited. Based on the present study it cannot be stated if the post-exercise cTnI increases were pathologically or physiologically in nature.

### Interpretation of exercise-induced cTn in athletes

Basal values of cTnT in football players were observed to be higher in healthy adults than children [21]. Likewise, the 99^th^ percentile URL seems to increase with age and to be higher in men [33]. Nevertheless, according to a recent meta-analysis the modifying effect of age on pre- and post-exercise TnI is unclear [11]. Although the authors found the 99th percentile for high-sensitive cTnT for male athletes at baseline and after running reduced with age, a clear effect of age with true substantial magnitude was demonstrated to be unlikely [11]. In the present study elevated cardiac biomarkers were not associated with CVRF and cardiovascular diseases. This observation emphasizes the importance of correctly interpreting pre- and post-exercise cTnI values in older athletes to avoid overdiagnosis of potential myocardial damage. It has been shown that a cTnT increase was attenuated 4.5 hours after exercise testing in patients with evidence of reversible myocardial ischemia compared to patients without ischaemia, suggesting that mechanisms other than ischemia are associated with this phenomenon [34]. Nevertheless, pre-existing cardiovascular diseases and risk factors in older athletes should be taking into consideration when interpreting post-exercise cTn values [35]. Although traditional CVRF were not associated with post-exercise cTn increase in a small group of middle-aged marathon runners, [36] a study in older athletes found an association between cardiovascular pathology and increased cTn values following prolonged moderate walking [37]. Since the veteran football players in the present study represented a vulnerable population with a considerable number of CVRF and/or pre-existing cardiovascular diseases, we did not adjust the cut-off value for myocardial damage as would have been appropriate for exclusively healthy athletes (to avoid many false cTn-positive results). Available evidence for the underlying mechanisms of exercise-induced cTn suggests increases in cardiomyocyte turnover and membrane permeability (reversible injury: cell wounds, extracellular blebs, exocytosis) [38, 39]. The extent to which other mechanisms such as increased rates of apoptosis and necrosis (irreversible damage: microdamage) are involved is still unclear [38]. Based on cellular level, it was beyond the scope of this study to determine whether the cTnI elevations after exercise are indicative of cardiomyocyte injury or damage. However, acute myocardial damage seemed unlikely. The absence of cardiac symptoms, both during the football match and stress test, an unremarkable exercise ECG and echocardiogram in 100% and > 90% of the cTnI-positive players, respectively, suggest a high proportion of physiological cTnI elevations.

### Methodological considerations and limitations

The strength of this study is that for the first time, the response of exercise-induced cTnI and BNP to football (and high-intensity intermittent exercise in general) has been investigated in a large group of older athletes in competition and that the cardiovascular risk in this potentially vulnerable population, measured by cardiac biomarkers, has been assessed by comprehensive cardiac follow-up. Compared with previous studies on exercise-induced cTn, cardiac follow-up was rarely performed, and our sample size was in the upper range. Nevertheless, some limitations remain to be addressed. Only 62 of 112 players (55%) followed our invitation for a cardiac follow-up, however, a large proportion of cTnI-positive players were represented (31 of 38 players, 82%). Furthermore, the exercise load during a football match could not be precisely determined. ECG vests were used, but unfortunately the supplier company responsible went bankrupt during the study period and data (stored in a cloud) could not be analysed at the end of the study. This also affected a previously published study [40]. Nevertheless, cardiac stress during a veterans’ football match has already been reported to be high [7]. A third limitation is that substitutions during match were allowed as often as desired (intending to ensure high external validity) potentially contributing to different cTnI response, although the magnitude of individual cTn release is extremely variable, even after the same exercise [41].

Another limitation was that we did not perform repeated blood sampling up to 24 hours post-match to better understand the kinetics of cardiac biomarkers in veteran football players. Diagnostically, repeated measurements post-exercise would have been ideal, but in addition to logistical problems, would probably also have led to higher non-adherence to the study protocol (more visits). The study was designed to assess the cardiovascular risk in veteran players via follow up (specifically, how much and which diseases are present), not to soley investigate post-exercise cTnI kinetics. Nevertheless, the cardiological follow-up made a diagnostic evaluation of the cTnI-positive players possible. Future studies should correlate post-exercise cardiac biomarker courses with training load metrics of a veterans`football match, to better classify the clinical relevance of temporary elevations.

## Conclusion

Veteran football players appear not to be exposed to overly cardiac stress compared with other sports. However, 6 of 62 players screened (10%) had relevant cardiac diseases, some of which may be associated with an increased risk of sudden cardiac death during sports. Some of these players would even be disqualified from organized competitive sports according to current recommendations [42]. However, in such cases, an informed individual decision together with the player including appropriate documentation and, possible consultation with an experienced sports cardiologist is considered desirable.

Since the proportion of relevant cardiac diseases or abnormalities in this study was 10%, it is recommended that veteran football players undergo regular (about once per year) sports cardiological examinations including history, physical examination, resting ECG, and echocardiography. An exercise ECG is recommended at least for objective assessment of the maximal workload. Whether this examination in veteran football players can increase the sensitivity to detect (unknown and asymptomatic) cardiac disease cannot be assessed based on the present data. Measurement of exercise-induced cTnI and BNP does not seem to contribute to an increased sensitivity or specificity in the detection of cardiac disease in veteran football players.

## Supporting information

S1 Table Baseline characteristics of cTnI-positive and cTnI-negative veteran football players. (PDF)

S1 Data Tabular presentation of all relevant data. (PDF)
