# Supplementary material for: Cardiovascular risk of veterans’ football: An observational cohort study with follow-up
Source: PLoS One. 2024 Apr 5;19(4):e0297951. doi: 10.1371/journal.pone.0297951 (PMC10997130; doi:10.1371/journal.pone.0297951)
Supplement: S1 Table — (PDF) [file pone.0297951.s001.pdf]

Baseline characteristics of cTnI-positive and cTnI-negative veteran football players

	cTnI-positive	cTnI-negative	p
**n**	**31**	**31**	
Age [years]	53.0 ± 8.7	56.7 ± 5.6	0.13
Height [cm]	175.1 ± 8.3	177.0 ± 6.4	0.27
Weight [kg]	83.6 ± 12.5	84.2 ± 9.9	0.73
BMI [kg/m^2]	27.2 ± 3.2	26.9 ± 3.0	0.70
**Cardiovascular risk factors, n (%)**			
Type 2 diabetes	1 (3)	1 (3)	0.94
Hypertension	10 (32)	4 (13)	0.13
Hypercholesterolemia	10 (32)	5 (16)	0.20
Smoking	15 (48)	9 (29)	0.05
Obesity	7 (23)	4 (13)	0.40
Family history	10 (32)	8 (26)	0.20
**Number of cardiovascular risk factors, n (%)**			
0	8 (24)	13 (42)	0.11
1	10 (32)	12 (39)	0.96
2	3 (9)	3 (10)	0.90
3	4 (12)	2 (6)	0.67
4	6 (18)	1 (3)	0.10
**Heart disease, n (%)**			
Coronary artery disease	1 (3)	2 (6)	0.62
Patent foramen ovale	1 (3)	-	-
Myocarditis	1 (3)	-	-
Coronary artery anomaly (muscle bridge)	1 (3)	-	-
**Medication, n (%)**			
Aspirin or clopidogrel	1 (3)	3 (10)	0.34
Antihypertensive agents	5 (15)	3 (10)	0.71
Hypoglycemic agents	2 (6)	1 (3)	0.62
Beta-blocker	-	2 (6)	-
Statins	2 (6)	4 (13)	0.41

*Plus-minus values are means ± standard deviation. Alternatively, in cases of skewed distribution data are presented as medians and interquartile range.
