# Supplementary material for: Cardiovascular risk of veterans’ football: An observational cohort study with follow-up
Source: PLoS One. 2024 Apr 5;19(4):e0297951. doi: 10.1371/journal.pone.0297951 (PMC10997130; doi:10.1371/journal.pone.0297951)
Supplement: S1 Data — (PDF) [file pone.0297951.s002.pdf]

Baseline characteristics

n	age [y]	height [cm]	weight [kg]	BMI [kg/m^2]	CVRF (n)	FH (n)	HCL (n)	HTN (n)	SM (n)	T2D (n)	OB (n)	CAD (n)	MC (n)	AR (n)	HOCM (n)	AS (n)	CS (n)
1	51	173	74	24,7	1	0	0	0	1	0	0	0	0	0	0	0	0
2	51	179	80	25,0	0	0	0	0	0	0	0	0	0	0	0	0	0
3	50	178	75	23,7	0	0	0	0	0	0	0	0	0	0	0	0	0
4	49	173	77	25,7	0	0	0	0	0	0	0	0	0	0	0	0	0
5	58	180	80	24,7	0	0	0	0	0	0	0	0	0	0	0	0	0
6	33	178	88	27,8	1	0	0	0	1	0	0	0	0	0	0	0	0
7	54	179	78	24,3	4	1	1	1	1	0	0	1	1	0	0	0	0
8	52	163	75	28,2	1	0	0	0	1	0	0	0	0	0	0	0	0
9	61	180	80	24,7	1	0	0	1	0	0	0	0	0	0	0	0	0
10	51	180	84	25,9	1	0	0	0	1	0	0	0	0	0	0	0	0
11	51	172	75	25,4	0	0	0	0	0	0	0	0	0	0	0	0	0
12	51	174	74	24,4	2	0	0	1	1	0	0	1	0	0	0	0	0
13	40	182	115	34,7	4	0	0	1	1	1	1	0	0	0	0	0	0
14	66	180	89	27,5	4	0	1	1	1	1	0	0	0	0	0	0	0
15	48	183	90	26,9	3	0	1	0	1	1	0	0	0	0	0	0	0
16	36	180	70	21,6	1	0	0	0	0	1	0	0	0	0	0	0	0
17	53	178	95	30,0	2	0	0	0	1	1	0	0	0	0	0	0	0
18	51	181	80	24,4	0	0	0	0	0	0	0	0	0	0	0	0	0
19	44	176	85	27,4	3	1	0	1	1	0	0	0	0	0	0	0	0
20	40	180	89	27,5	1	0	0	0	1	0	0	0	0	1	0	0	0
21	52	166	68	24,7	1	0	0	0	1	0	0	0	0	0	0	0	0
22	77	176	90	29,1	4	0	1	1	1	1	0	0	0	0	0	1	0
23	66	165	68	25,0	0	0	0	0	0	0	0	0	0	0	0	0	0
24	71	171	72	24,6	1	0	0	1	0	0	0	0	0	0	0	0	0
25	34	198	113	28,8	2	0	0	1	1	0	0	0	0	0	0	0	0
26	55	178	81	25,6	0	0	0	0	0	0	0	0	0	0	0	0	0
27	56	178	118	37,2	2	0	0	0	1	0	1	0	0	0	0	0	0

CVRF, cardiovascular risk factors; FH, family history; HCL, hypercholesterolemia; HTN, hypertension; SM, smoking (current and former smokers combined); py, pack years; T2D, type 2 diabetes; OB, obesity; CAD, coronary artery disease; MC, myocarditis in the past; AR, arrhythmia; HOCM, hypertrophic obstructive cardiomyopathy; AS, aortic stenosis; CS, carotid artery stenosis.

Baseline characteristics

n	age [y]	height [cm]	weight [kg]	BMI [kg/m²]	CVRF (n)	FH (n)	HCL (n)	HTN (n)	SM (n)	T2D (n)	OB (n)	CAD (n)	MC (n)	AR (n)	HOCM (n)	AS (n)	CS (n)
28	54	167	86	30,8	2	0	0	0	1	0	1	0	0	0	0	0	0
29	72	170	71	24,6	2	0	1	1	0	0	0	1	0	0	0	0	0
30	53	179	74	23,1	1	1	0	0	0	0	0	0	0	0	0	0	0
31	53	178	83	26,2	1	1	0	0	0	0	0	0	0	0	0	0	0
32	54	182	90	27,2	0	0	0	0	0	0	0	0	0	0	0	0	0
33	46	180	106	32,7	3	0	0	1	1	0	1	1	0	0	0	0	0
34	45	180	98	30,2	4	0	1	1	1	0	1	1	0	0	0	0	0
35	51	181	85	25,9	3	1	1	1	0	0	0	0	0	0	0	0	1
36	51	175	74	24,2	1	1	0	0	0	0	0	0	0	0	0	0	0
37	39	171	79	27,0	1	0	0	0	1	0	0	0	0	0	0	0	0
38	53	170	66	22,8	0	0	0	0	0	0	0	0	0	0	0	0	0
39	49	175	80	26,1	1	0	1	0	0	0	0	0	0	0	0	0	0
40	62	184	80	23,6	1	0	0	0	1	0	0	0	0	0	0	0	0
41	35	170	65	22,5	0	0	0	0	0	0	0	0	0	0	0	0	0
42	52	192	98	26,6	0	0	0	0	0	0	0	0	0	0	0	0	0
43	44	170	70	24,2	1	0	0	0	1	0	0	0	1	0	0	0	0
44	31	184	110	32,5	2	0	0	0	1	0	1	0	0	0	0	0	0
45	37	180	75	23,1	0	0	0	0	0	0	0	0	0	0	0	0	0
46	52	173	83	27,7	0	0	0	0	0	0	0	0	0	1	0	0	0
47	56	188	80	22,6	3	1	1	0	1	0	0	1	0	0	1	0	0
48	54	176	77	24,9	1	0	0	0	1	0	0	0	0	0	0	0	0
49	46	167	72	25,8	2	1	1	0	0	0	0	0	0	0	0	0	0
50	51	168	79	28,0	0	0	0	0	0	0	0	0	0	0	0	0	0
51	73	163	75	28,2	3	0	1	1	1	0	0	0	0	0	0	0	0
52	44	187	105	30,0	3	1	0	0	1	0	1	0	0	0	0	0	0
53	45	172	70	23,7	1	1	0	0	0	0	0	0	0	0	0	0	0
54	44	180	100	30,9	2	0	0	0	1	0	1	0	0	0	0	0	0

CVRF, cardiovascular risk factors; FH, family history; HCL, hypercholesterolemia; HTN, hypertension; SM, smoking (current and former smokers combined); py, pack years; T2D, type 2 diabetes; OB, obesity; CAD, coronary artery disease; MC, myocarditis in the past; AR, arrhythmia; HOCM, hypertrophic obstructive cardiomyopathy; AS, aortic stenosis; CS, carotid artery stenosis.

Baseline characteristics

n	age [y]	height [cm]	weight [kg]	BMI [kg/m^2]	CVRF (n)	FH (n)	HCL (n)	HTN (n)	SM (n)	T2D (n)	OB (n)	CAD (n)	MC (n)	AR (n)	HOCM (n)	AS (n)	CS (n)
55	51	175	78	25,5	0	0	0	0	0	0	0	0	0	0	0	0	0
56	50	182	105	31,7	3	1	0	0	1	0	1	1	0	0	0	0	0
57	71	163	60	22,6	1	0	0	0	1	0	0	0	0	0	0	0	0
58	49	170	70	24,2	1	1	0	0	0	0	0	0	0	0	0	0	0
59	74	172	87	29,4	0	0	0	0	0	0	0	0	0	0	0	0	0
60	45	164	76	28,3	1	0	0	0	1	0	0	0	0	0	0	0	0
61	42	170	74	25,6	0	0	0	0	0	0	0	0	0	0	0	0	0
62	49	185	85	24,8	1	0	0	0	1	0	0	0	0	0	0	0	0
63	51	189	91	25,5	0	0	0	0	0	0	0	0	0	0	0	0	0
64	57	182	90	27,2	0	0	0	0	0	0	0	0	0	0	0	0	0
65	48	188	90	25,5	0	0	0	0	0	0	0	0	0	0	0	0	0
66	47	170	82	28,4	0	0	0	0	0	0	0	0	0	0	0	0	0
67	36	190	93	25,8	1	0	0	0	1	0	0	0	0	0	0	0	0
68	47	177	80	25,5	3	0	1	0	1	1	0	0	0	0	0	0	0
69	56	164	79	29,2	1	0	0	0	0	1	0	0	0	0	0	0	0
70	57	170	86	26,0	1	1	0	0	0	0	0	0	1	0	0	0	0
71	57	170	87	28,4	1	0	0	0	0	1	0	0	0	0	0	0	0
72	58	170	87	24,2	2	0	0	0	1	1	0	0	0	0	0	0	0
73	43	182	87	30,2	1	0	0	0	0	0	1	0	0	0	0	0	0
74	54	180	88	25,6	3	1	1	0	1	0	0	0	0	1	0	0	0
75	55	172	88	25,7	1	1	0	0	0	0	0	0	0	0	0	0	0
76	41	182	89	28,1	1	0	0	0	1	0	0	0	0	0	0	0	0
77	66	179	89	27,2	1	0	1	0	0	0	0	0	0	0	0	0	0
78	55	178	90	25,2	0	0	0	0	0	0	0	0	0	0	0	0	0
79	64	176	90	24,2	1	0	1	0	0	0	0	0	0	0	0	0	0
80	32	175	90	25,8	1	0	0	0	1	0	0	0	0	0	0	0	0
81	50	173	91	33,1	4	0	1	1	1	0	1	0	0	0	0	0	0

CVRF, cardiovascular risk factors; FH, family history; HCL, hypercholesterolemia; HTN, hypertension; SM, smoking (current and former smokers combined); py, pack years; T2D, type 2 diabetes; OB, obesity; CAD, coronary artery disease; MC, myocarditis in the past; AR, arrhythmia; HOCM, hypertrophic obstructive cardiomyopathy; AS, aortic stenosis; CS, carotid artery stenosis.

Baseline characteristics

n	age [y]	height [cm]	weight [kg]	BMI $[kg/m^2]$	CVRF (n)	FH (n)	HCL (n)	HTN (n)	SM (n)	T2D (n)	OB (n)	CAD (n)	MC (n)	AR (n)	HOCM (n)	AS (n)	CS (n)
82	52	177	70	22,3	0	0	0	0	0	0	0	0	0	0	0	0	0
83	44	179	85	26,5	1	0	0	0	1	0	0	0	0	0	0	0	0
84	38	171	78	26,7	0	0	0	0	0	0	0	0	0	0	0	0	0
85	68	172	71	24,0	1	0	0	0	1	0	0	0	0	0	0	0	0
86	53	180	95	29,3	1	0	0	0	1	0	0	0	0	0	0	0	0
87	50	172	88	29,7	0	0	0	0	0	0	0	0	0	0	0	0	0
88	47	178	80	25,2	0	0	0	0	0	0	0	0	0	0	0	0	0
89	34	173	75	25,1	0	0	0	0	0	0	0	0	0	0	0	0	0
90	47	171	80	27,4	0	0	0	0	0	0	0	0	0	0	0	0	0
91	53	166	79	28,7	1	0	1	0	0	0	0	0	0	0	0	0	0
92	35	180	86	26,5	0	0	0	0	0	0	0	0	0	0	0	0	0
93	53	168	72	25,5	0	0	0	0	0	0	0	0	0	0	0	0	0
94	47	178	83	26,2	1	0	0	0	1	0	0	0	0	0	0	0	0
95	53	173	85	28,4	2	0	1	0	1	0	0	0	0	0	0	0	0
96	60	189	89	24,9	2	0	0	1	1	0	0	0	0	0	0	0	0
97	50	180	78	24,1	1	0	0	0	1	0	0	0	0	0	0	0	0
98	51	179	84	26,2	1	1	0	0	0	0	0	0	0	0	0	0	0
99	74	182	82	24,8	1	0	0	0	1	0	0	0	0	0	0	0	0
100	45	186	84	24,3	0	0	0	0	0	0	0	0	0	0	0	0	0
101	46	181	93	28,4	2	1	0	0	1	0	0	0	0	0	0	0	0
102	50	175	88	28,7	2	1	1	0	0	0	0	0	0	0	0	0	0
103	49	166	69	25,0	1	0	0	0	1	0	0	0	0	0	0	0	0
104	76	172	75	25,4	0	0	0	0	0	0	0	0	0	0	0	0	0
105	47	172	82	27,7	0	0	0	0	0	0	0	0	0	0	0	0	0
106	54	178	95	30,0	1	0	0	0	0	0	1	0	0	0	0	0	0
107	52	188	84	23,8	0	0	0	0	0	0	0	0	0	0	0	0	0
108	53	176	74	23,9	1	0	1	0	0	0	0	0	0	0	0	0	0

CVRF, cardiovascular risk factors; FH, family history; HCL, hypercholesterolemia; HTN, hypertension; SM, smoking (current and former smokers combined); py, pack years; T2D, type 2 diabetes; OB, obesity; CAD, coronary artery disease; MC, myocarditis in the past; AR, arrhythmia; HOCM, hypertrophic obstructive cardiomyopathy; AS, aortic stenosis; CS, carotid artery stenosis.

Baseline characteristics

n	age [y]	height [cm]	weight [kg]	BMI [kg/m^2]	CVRF (n)	FH (n)	HCL (n)	HTN (n)	SM (n)	T2D (n)	OB (n)	CAD (n)	MC (n)	AR (n)	HOCM (n)	AS (n)	CS (n)
109	46	185	82	24.0	0	0	0	0	0	0	0	0	0	0	0	0	0
110	78	165	69	25.3	0	0	0	0	0	0	0	0	0	0	0	0	0
111	54	178	89	28.1	1	0	0	1	0	0	0	0	0	0	0	0	0
112	49	175	85	27.8	0	0	0	0	0	0	0	0	0	0	0	0	0

CVRF, cardiovascular risk factors; FH, family history; HCL, hypercholesterolemia; HTN, hypertension; SM, smoking (current and former smokers combined); py, pack years; T2D, type 2 diabetes; OB, obesity; CAD, coronary artery disease; MC, myocarditis in the past; AR, arrhythmia; HOCM, hypertrophic obstructive cardiomyopathy; AS, aortic stenosis; CS, carotid artery stenosis.

Baseline characteristics

n	CVD (n)	MED (n)	APA (n)	OAC (n)	ACE (n)	RAI (n)	BB (n)	DIU (n)	STA (n)	MET (n)	AA (n)	LTY (n)
1	0	0	0	0	0	0	0	0	0	0	0	0
2	0	0	0	0	0	0	0	0	0	0	0	0
3	0	0	0	0	0	0	0	0	0	0	0	0
4	0	0	0	0	0	0	0	0	0	0	0	0
5	0	0	0	0	0	0	0	0	0	0	0	0
6	0	1	1	0	1	0	1	0	1	0	0	0
7	2	0	0	0	0	0	0	0	0	0	0	0
8	0	0	0	0	0	0	0	0	0	0	0	0
9	0	0	0	0	0	0	0	0	0	0	0	0
10	0	0	0	0	0	0	0	0	0	0	0	0
11	0	0	0	0	0	0	0	0	0	0	0	0
12	0	1	0	0	1	0	0	0	0	0	0	0
13	0	1	0	0	1	0	0	0	0	1	0	0
14	0	0	0	0	0	0	0	0	0	0	0	0
15	0	0	0	0	0	0	0	0	0	0	0	0
16	0	0	0	0	0	0	0	0	0	0	0	0
17	1	1	1	0	0	0	0	0	0	0	0	0
18	0	0	0	0	0	0	0	0	0	0	0	0
19	0	1	0	0	1	0	0	0	0	0	0	0
20	1	0	0	0	0	0	0	0	0	0	0	0
21	0	0	0	0	0	0	0	0	0	0	0	0
22	1	1	0	0	0	1	0	0	1	0	0	0
23	0	1	1	0	0	0	0	0	0	0	0	0
24	0	0	0	0	0	0	0	0	0	0	0	0
25	0	1	0	0	1	0	0	0	0	0	0	0
26	0	0	0	0	0	0	0	0	0	0	0	0
27	0	0	0	0	0	0	0	0	0	0	0	0

CVD, Cardiovascular disease; MED, Medication; APA, Antiplatelet agent (aspirin or clopidogrel); OAC, Oral anticoagulant; ACE, ACE inhibitor; RAI, Renin-angiotensin inhibitor, BB, Beta-blocker; DIU, Diuretics; STA, Statins; MET, Metformin, AA, Antiarrhythmics; LTY, L-Thyroxin.

Baseline characteristics

n	CVD (n)	MED (n)	APA (n)	OAC (n)	ACE (n)	RAI (n)	BB (n)	DIU (n)	STA (n)	MET (n)	AA (n)	LTY (n)
28	0	0	0	0	0	0	0	0	0	0	0	0
29	1	1	0	1	0	0	0	0	1	0	0	0
30	0	0	0	0	0	0	0	0	0	0	0	0
31	0	0	0	0	0	0	0	0	0	0	0	0
32	1	0	0	0	0	0	0	0	0	0	0	0
33	1	1	0	0	0	0	1	0	0	0	0	0
34	1	1	1	0	1	0	0	0	1	0	0	0
35	1	1	1	0	0	1	0	0	1	0	0	0
36	0	0	0	0	0	0	0	0	0	0	0	0
37	0	0	0	0	0	0	0	0	0	0	0	0
38	0	0	0	0	0	0	0	0	0	0	0	0
39	0	0	0	0	0	0	0	0	0	0	0	0
40	0	0	0	0	0	0	0	0	0	0	0	0
41	0	0	0	0	0	0	0	0	0	0	0	0
42	0	0	0	0	0	0	0	0	0	0	0	0
43	1	0	0	0	0	0	0	0	0	0	0	0
44	0	0	0	0	0	0	0	0	0	0	0	0
45	0	0	0	0	0	0	0	0	0	0	0	0
46	1	0	0	0	0	0	0	0	0	0	0	0
47	2	1	0	0	0	0	0	0	0	0	1	0
48	0	0	0	0	0	0	0	0	0	0	0	0
49	0	0	0	0	0	0	0	0	0	0	0	0
50	0	0	0	0	0	0	0	0	0	0	0	0
51	0	1	0	0	0	1	0	0	1	0	0	0
52	1	0	0	0	0	0	0	0	0	0	0	1
53	0	0	0	0	0	0	0	0	0	0	0	0
54	0	0	0	0	0	0	0	0	0	0	0	0

CVD, Cardiovascular disease; MED, Medication; APA, Antiplatelet agent (aspirin or clopidogrel); OAC, Oral anticoagulant; ACE, ACE inhibitor; RAI, Renin-angiotensin inhibitor, BB, Beta-blocker; DIU, Diuretics; STA, Statins; MET, Metformin, AA, Antiarrhythmics; LTY, L-Thyroxin.

Baseline characteristics

n	CVD (n)	MED (n)	APA (n)	OAC (n)	ACE (n)	RAI (n)	BB (n)	DIU (n)	STA (n)	MET (n)	AA (n)	LTY (n)
55	0	1	0	0	0	0	0	0	0	0	0	1
56	0	0	0	0	0	0	0	0	0	0	0	0
57	0	0	0	0	0	0	0	0	0	0	0	0
58	0	0	0	0	0	0	0	0	0	0	0	0
59	0	0	0	0	0	0	0	0	0	0	0	0
60	0	0	0	0	0	0	0	0	0	0	0	0
61	0	0	0	0	0	0	0	0	0	0	0	0
62	0	0	0	0	0	0	0	0	0	0	0	0
63	0	0	0	0	0	0	0	0	0	0	0	0
64	0	0	0	0	0	0	0	0	0	0	0	0
65	0	1	0	0	0	0	0	0	0	0	0	1
66	0	0	0	0	0	0	0	0	0	0	0	0
67	0	0	0	0	0	0	0	0	0	0	0	0
68	0	1	0	0	0	0	0	0	1	1	0	0
69	0	1	0	0	0	0	0	0	0	1	0	0
70	1	0	0	0	0	0	0	0	0	0	0	0
71	0	0	0	0	0	0	0	0	0	0	0	0
72	0	1	0	0	0	0	0	0	0	1	0	0
73	0	0	0	0	0	0	0	0	0	0	0	0
74	1	1	0	0	0	0	0	0	1	0	0	0
75	0	0	0	0	0	0	0	0	0	0	0	0
76	0	0	0	0	0	0	0	0	0	0	0	0
77	0	0	0	0	0	0	0	0	0	0	0	0
78	0	0	0	0	0	0	0	0	0	0	0	0
79	0	1	1	0	0	0	0	0	1	0	0	0
80	0	0	0	0	0	0	0	0	0	0	0	0
81	0	1	0	0	1	0	0	0	1	0	0	0

CVD, Cardiovascular disease; MED, Medication; APA, Antiplatelet agent (aspirin or clopidogrel); OAC, Oral anticoagulant; ACE, ACE inhibitor; RAI, Renin-angiotensin inhibitor, BB, Beta-blocker; DIU, Diuretics; STA, Statins; MET, Metformin, AA, Antiarrhythmics; LTY, L-Thyroxin.

Baseline characteristics

n	CVD (n)	MED (n)	APA (n)	OAC (n)	ACE (n)	RAI (n)	BB (n)	DIU (n)	STA (n)	MET (n)	AA (n)	LTY (n)
82	0	0	0	0	0	0	0	0	0	0	0	0
83	0	0	0	0	0	0	0	0	0	0	0	0
84	0	0	0	0	0	0	0	0	0	0	0	0
85	0	0	0	0	0	0	0	0	0	0	0	0
86	0	0	0	0	0	0	0	0	0	0	0	0
87	0	0	0	0	0	0	0	0	0	0	0	0
88	0	0	0	0	0	0	0	0	0	0	0	0
89	0	0	0	0	0	0	0	0	0	0	0	0
90	0	0	0	0	0	0	0	0	0	0	0	0
91	0	0	0	0	0	0	0	0	0	0	0	0
92	0	0	0	0	0	0	0	0	0	0	0	0
93	0	0	0	0	0	0	0	0	0	0	0	0
94	0	0	0	0	0	0	0	0	0	0	0	0
95	0	1	1	0	0	0	0	0	0	0	0	0
96	0	1	0	0	1	0	0	1	0	0	0	0
97	0	1	0	0	0	0	0	0	0	0	0	1
98	0	0	0	0	0	0	0	0	0	0	0	0
99	0	0	0	0	0	0	0	0	0	0	0	0
100	0	0	0	0	0	0	0	0	0	0	0	0
101	0	0	0	0	0	0	0	0	0	0	0	0
102	0	0	0	0	0	0	0	0	0	0	0	0
103	0	0	0	0	0	0	0	0	0	0	0	0
104	0	0	0	0	0	0	0	0	0	0	0	0
105	0	0	0	0	0	0	0	0	0	0	0	0
106	0	0	0	0	0	0	0	0	0	0	0	0
107	0	0	0	0	0	0	0	0	0	0	0	0
108	0	0	0	0	0	0	0	0	0	0	0	0

CVD, Cardiovascular disease; MED, Medication; APA, Antiplatelet agent (aspirin or clopidogrel); OAC, Oral anticoagulant; ACE, ACE inhibitor; RAI, Renin-angiotensin inhibitor, BB, Beta-blocker; DIU, Diuretics; STA, Statins; MET, Metformin, AA, Antiarrhythmics; LTY, L-Thyroxin.

Baseline characteristics

n	CVD (n)	MED (n)	APA (n)	OAC (n)	ACE (n)	RAI (n)	BB (n)	DIU (n)	STA (n)	MET (n)	AA (n)	LTY (n)
109	0	0	0	0	0	0	0	0	0	0	0	0
110	0	1	0	0	1	0	0	0	0	0	0	0
111	0	0	0	0	0	0	0	0	0	0	0	0
112	0	0	0	0	0	0	0	0	0	0	0	0

CVD, Cardiovascular disease; MED, Medication; APA, Antiplatelet agent (aspirin or clopidogrel); OAC, Oral anticoagulant; ACE, ACE inhibitor; RAI, Renin-angiotensin inhibitor, BB, Beta-blocker; DIU, Diuretics; STA, Statins; MET, Metformin, AA, Antiarrhythmics; LTY, L-Thyroxin.

Blood parameters (pre-match)

n	ECC [million/mm³]	HB [g/dl]	HKT [%]	WCC [thousand/mm³]	PC [thousand/ mm³]	CK [U/L]	Urea [U/L]	CR [U/L]	cTnI [ng/L]	BNP [pg/ml]
1	5,0	15,6	46,2	5,0	272	190	38	0,89	0	32
2	4,8	15,3	46,8	6,3	173	75	32	0,97	0	14
3	5,0	14,5	44,9	6,4	250	104	41	1,00	0	54
4	4,6	14,8	44,6	6,5	196	68	44	0,93	0	18
5	4,5	13,7	41,6	7,6	363	139	34	0,87	0	17
6	5,2	16,4	47,8	8,7	239	231	42	0,88	31	20
7	4,7	13,4	41,7	7,6	343	208	35	1,13	0	14
8	4,9	13,4	42,2	8,2	295	198	26	0,90	6	38
9	4,5	13,7	40,3	7,6	338	210	37	0,97	0	24
10	4,4	15,1	45,2	5,0	287	99	37	0,78	2	42
11	4,3	13,8	41,2	5,9	239	383	49	1,13	0	17
12	4,9	14,8	43,4	7,6	268	309	32	1,14	28	41
13	4,7	15,7	46,6	9,6	292	289	35	1,25	0	19
14	5,5	15,1	45,0	5,7	235	149	36	1,05	0	69
15	5,5	15,9	46,8	4,6	191	84	29	1,00	0	25
16	5,3	16,6	49,5	7,7	250	305	26	0,88	2	18
17	5,1	16,4	47,1	12,3	277	101	49	0,71	5	61
18	4,4	13,9	42,3	7,9	299	122	35	1,16	0	30
19	4,7	14,3	42,6	9,0	306	198	39	1,00	10	17
20	5,1	16,0	46,9	8,3	219	91	27	0,81	0	27
21	5,0	16,2	47,1	9,2	323	102	40	0,85	0	89
22	4,6	13,9	41,6	7,0	214	162	43	1,15	0	78
23	4,2	13,3	39,3	7,3	177	125	37	1,16	0	52
24	4,2	13,2	40,5	4,9	228	578	36	0,93	0	79
25	5,1	16,6	49,0	8,9	248	262	32	1,27	0	25
26	5,4	16,6	50,7	7,3	246	250	46	0,90	27	33
27	4,9	15,0	45,9	8,0	207	436	34	1,11	0	33

ECC, erythrocyte count; HB, hemoglobin; HKT, hematocrit; WCC, white-cell count; PCC, platelet count; CK, creatine kinase; CR, creatinine; cTnI, cardiac troponin I; BNP, B-type natriuretic peptide.

Blood parameters (pre-match)

n	ECC [million/mm^3]	HB [g/dl]	HKT [%]	WCC [thousand/mm^3]	PC [thousand/ mm^3]	CK [U/L]	Urea [U/L]	CR [U/L]	cTnI [ng/L]	BNP [pg/ml]
28	5,1	15,7	46,1	5,5	361	235	24	1,11	1	23
29	4,9	14,4	43,7	8,0	258	175	54	0,98	0	282
30	4,8	15,0	45,4	4,2	181	364	41	1,10	0	38
31	4,2	13,7	42,3	6,8	260	190	56	1,11	0	24
32	4,2	13,6	40,2	6,2	378	1150	60	1,11	0	27
33	4,7	14,8	44,7	8,5	228	266	42	1,01	0	53
34	4,5	14,6	43,8	5,8	271	262	54	1,05	0	31
35	4,6	13,2	41,3	5,4	223	141	44	0,81	4	51
36	5,0	15,0	44,5	5,4	209	116	38	1,37	0	42
37	5,0	15,9	46,9	4,7	260	181	28	1,20	0	15
38	4,8	15,0	44,8	5,2	231	116	25	0,78	0	15
39	5,0	14,7	44,4	6,0	211	136	41	0,85	0	19
40	4,8	15,7	45,7	5,6	295	136	34	0,94	0	43
41	5,6	16,5	48,3	7,7	259	184	43	0,99	0	32
42	4,7	13,7	41,1	8,3	257	420	28	1,06	0	36
43	4,7	14,7	44,6	10,0	304	154	28	1,05	1	179
44	4,8	15,2	44,4	7,5	226	143	20	0,98	0	17
45	5,1	15,0	44,0	5,4	185	144	40	0,71	0	34
46	4,0	13,7	40,9	4,9	189	61	44	0,83	0	46
47	4,5	14,7	44,3	13,9	114	238	40	0,89	18	52
48	5,0	15,2	44,6	6,1	322	128	36	1,28	0	25
49	4,9	14,3	42,4	4,9	258	190	30	0,98	0	16
50	5,3	14,6	43,2	7,3	212	123	36	0,90	12	25
51	4,3	14,1	40,8	6,3	142	48	34	1,08	0	29
52	4,7	14,4	43,7	6,6	250	145	39	1,04	0	34
53	4,6	14,3	42,6	8,6	334	271	28	0,92	0	25
54	5,0	14,8	45,2	6,7	229	204	33	0,99	2	31

ECC, erythrocyte count; HB, hemoglobin; HKT, hematocrit; WCC, white-cell count; PCC, platelet count; CK, creatine kinase; CR, creatinine; cTnI, cardiac troponin I; BNP, B-type natriuretic peptide.

Blood parameters (pre-match)

n	ECC [million/mm^3]	HB [g/dl]	HKT [%]	WCC [thousand/mm^3]	PC [thousand/ mm^3]	CK [U/L]	Urea [U/L]	CR [U/L]	cTnI [ng/L]	BNP [pg/ml]
55	4,8	14,6	44,7	8,2	307	54	32	1,15	0	23
56	4,8	14,8	44,0	7,6	258	440	44	1,12	5	21
57	4,9	15,5	45,4	7,0	378	59	40	1,11	1	89
58	4,8	14,4	43,0	5,2	251	211	46	0,85	1	17
59	4,7	15,1	44,6	5,9	225	37	42	0,89	0	34
60	4,8	15,7	46,5	8,4	348	176	51	1,07	2	22
61	4,6	14,1	41,2	6,4	323	243	30	0,79	0	41
62	4,5	15,4	44,5	10,3	284	106	38	0,88	0	25
63	4,9	14,7	43,6	4,8	193	284	39	0,94	0	21
64	4,7	14,6	43,1	6,9	307	118	44	0,89	0	80
65	4,6	14,0	42,2	5,3	269	210	38	0,99	0	78
66	5,0	15,0	43,3	5,4	225	101	33	0,92	0	19
67	4,6	13,9	42,0	5,0	275	124	36	0,82	0	24
68	4,7	15,0	45,3	6,5	244	92	31	0,85	0	15
69	4,4	13,6	40,0	5,7	266	143	29	0,87	0	18
70	5,1	15,8	48,3	10,1	276	108	31	1,03	0	30
71	4,5	13,6	40,1	7,5	264	984	31	1,03	0	31
72	4,4	13,8	40,8	6,1	275	251	39	1,02	13	32
73	5,4	16,4	47,0	9,2	289	192	38	0,99	28	22
74	5,1	15,7	45,8	6,5	231	154	42	1,11	28	14
75	4,7	15,1	44,6	5,8	265	154	34	0,93	0	27
76	5,0	15,2	45,5	6,1	165	134	43	0,98	0	19
77	4,8	14,5	43,0	4,3	253	58	37	0,91	0	42
78	4,3	13,7	40,5	7,6	308	146	33	0,94	31	21
79	4,8	14,3	43,3	8,6	260	75	43	0,76	0	53
80	4,9	15,1	45,3	12,1	211	433	23	1,04	0	23
81	4,8	15,1	45,5	7,9	239	346	34	1,00	6	28

ECC, erythrocyte count; HB, hemoglobin; HKT, hematocrit; WCC, white-cell count; PCC, platelet count; CK, creatine kinase; CR, creatinine; cTnI, cardiac troponin I; BNP, B-type natriuretic peptide.

Blood parameters (pre-match)

n	ECC [million/mm³]	HB [g/dl]	HKT [%]	WCC [thousand/mm³]	PC [thousand/ mm³]	CK [U/L]	Urea [U/L]	CR [U/L]	cTnI [ng/L]	BNP [pg/ml]
82	5,2	15,1	45,6	7,7	45,6	107	42	1,13	0	33
83	4,3	14,6	43,7	6,9	43,7	51	40	0,74	0	115
84	5,0	15,1	44,4	7,7	44,4	140	37	0,80	6	18
85	4,8	15,2	45,4	8,6	45,4	112	32	1,08	0	25
86	5,1	15,4	47,1	4,5	47,1	201	41	0,82	0	21
87	5,1	14,0	42,8	6,7	42,8	587	35	1,10	0	50
88	4,4	13,2	40,8	8,1	40,8	104	47	1,01	2	20
89	5,1	14,4	45,0	6,1	45,0	224	35	1,17	0	18
90	4,9	15,4	46,3	7,9	46,3	295	42	1,08	0	28
91	4,9	16,0	46,8	9,1	46,8	113	36	1,21	0	23
92	4,7	14,3	43,4	8,9	43,4	447	45	0,90	0	27
93	5,1	15,4	47,7	6,6	47,7	110	31	0,92	0	16
94	4,6	14,8	44,4	7,8	44,4	104	40	0,99	0	27
95	4,6	14,4	43,9	7,5	43,9	182	39	0,92	0	34
96	4,2	13,0	38,8	7,5	38,8	104	39	1,10	0	21
97	5,0	15,3	46,2	6,7	46,2	91	39	1,01	0	23
98	5,0	15,4	45,6	8,0	45,6	110	29	0,88	0	36
99	5,3	16,0	47,5	5,8	47,5	108	42	1,00	0	65
100	4,9	15,4	46,4	7,3	46,4	510	38	0,96	0	24
101	4,7	15,1	43,9	7,1	43,9	210	34	0,89	0	40
102	4,8	15,1	45,8	7,6	45,8	361	31	1,05	176	35
103	5,4	15,9	47,8	8,3	47,8	176	25	0,92	0	35
104	5,1	15,8	46,5	7,2	46,5	92	39	0,91	0	75
105	4,6	14,4	44,0	5,8	44,0	108	22	0,92	6	47
106	4,9	14,2	42,4	6,0	42,4	201	34	0,90	0	17
107	4,7	14,3	43,1	5,6	43,1	177	42	0,94	1	31
108	5,1	15,0	44,0	7,5	44,0	74	39	0,66	0	17

ECC, erythrocyte count; HB, hemoglobin; HKT, hematocrit; WCC, white-cell count; PCC, platelet count; CK, creatine kinase; CR, creatinine; cTnI, cardiac troponin I; BNP, B-type natriuretic peptide.

Blood parameters (pre-match)

n	ECC [million/mm^3]	HB [g/dl]	HKT [%]	WCC [thousand/mm^3]	PC [thousand/ mm^3]	CK [U/L]	Urea [U/L]	CR [U/L]	cTnI [ng/L]	BNP [pg/ml]
109	5,0	15,4	46,2	6,5	46,2	516	36	1,04	1	21
110	5,2	15,9	47,0	6,1	47,0	110	36	0,93	6	49
111	4,9	14,6	43,7	7,0	43,7	108	48	1,36	0	23
112	4,7	14,9	45,5	5,7	45,5	178	36	0,89	0	11

ECC, erythrocyte count; HB, hemoglobin; HKT, hematocrit; WCC, white-cell count; PCC, platelet count; CK, creatine kinase; CR, creatinine; cTnI, cardiac troponin I; BNP, B-type natriuretic peptide.

Blood parameters (post-match)

n	ECC [million/mm^3]	HB [g/dl]	HKT [%]	WCC [thousand/mm^3]	PC [thousand/ mm^3]	CK [U/L]	Urea [U/L]	CR [U/L]	cTnI [ng/L]	BNP [pg/ml]
1	5,1	15,9	46,9	5,9	312	235	41	1,27	0	59
2	5,1	16,3	48,4	10,3	216	113	34	1,24	0	29
3	4,5	13,4	40,6	8,6	261	135	46	1,31	0	49
4	4,6	14,7	43,9	10,3	240	88	48	1,22	0	20
5	4,3	13,3	39,8	9,5	363	180	34	1,09	0	22
6	5,0	15,9	45,8	9,1	248	297	45	0,97	41	23
7	4,9	13,9	42,6	7,8	381	268	37	1,42	0	25
8	5,0	14,0	43,6	9,0	318	285	31	1,10	1	53
9	4,6	14,1	41,2	7,6	370	253	36	1,06	0	26
10	4,2	14,5	41,7	6,1	319	185	38	1,00	4	46
11	4,5	14,2	42,4	7,6	277	436	54	1,32	0	33
12	5,0	14,9	43,6	7,1	270	404	33	1,39	8	42
13	4,7	15,6	45,4	9,4	297	408	39	1,54	10	24
14	5,4	14,9	44,4	6,3	238	174	37	1,30	0	80
15	5,4	15,7	45,9	7,3	229	127	34	1,16	1	45
16	5,1	16,0	46,5	11,2	290	430	29	1,45	4	49
17	4,8	15,5	44,6	13,2	281	134	52	0,80	36	47
18	4,3	13,6	39,5	10,4	285	170	39	1,37	0	53
19	4,7	14,4	42,7	12,3	380	267	47	1,32	0	22
20	5,1	15,8	45,8	9,2	234	135	30	1,09	4	40
21	4,8	15,3	44,8	8,4	319	160	48	0,99	3	30
22	4,7	14,1	41,7	8,7	255	197	42	1,32	0	101
23	4,0	12,5	36,5	5,2	174	150	40	1,34	11	44
24	4,1	12,8	38,1	6,3	260	665	38	1,25	0	107
25	4,9	16,0	46,0	9,9	246	286	32	1,59	26	28
26	5,3	16,1	47,6	9,4	222	304	52	1,15	32	38
27	4,7	14,7	43,8	7,7	206	516	34	1,37	0	40

ECC, erythrocyte count; HB, hemoglobin; HKT, hematocrit; WCC, white-cell count; PCC, platelet count; CK, creatine kinase; CR, creatinine; cTnI, cardiac troponin I; BNP, B-type natriuretic peptide.

Blood parameters (post-match)

n	ECC [million/mm^3]	HB [g/dl]	HKT [%]	WCC [thousand/mm^3]	PC [thousand/ mm^3]	CK [U/L]	Urea [U/L]	CR [U/L]	cTnI [ng/L]	BNP [pg/ml]
28	5,0	15,4	45,6	5,8	379	266	27	1,40	0	26
29	4,7	13,9	41,3	8,5	257	242	56	1,23	0	322
30	4,8	15,0	43,7	6,1	209	404	43	1,34	0	37
31	4,1	13,4	39,9	5,2	272	209	62	1,30	0	26
32	4,1	13,3	39,9	6,1	353	1297	62	1,07	0	35
33	4,6	14,7	42,7	11,4	216	367	43	1,33	0	43
34	4,6	14,5	43,8	5,8	276	281	54	0,90	0	33
35	4,5	13,2	40,0	6,8	236	187	51	1,64	0	53
36	4,7	14,5	41,8	6,0	211	219	40	1,38	12	47
37	5,1	16,2	47,0	8,9	324	221	29	1,04	0	25
38	4,6	14,2	41,7	8,6	208	147	32	1,01	5	17
39	4,9	14,4	43,6	5,8	207	192	42	1,26	0	21
40	4,8	15,9	47,0	7,0	284	157	31	0,97	0	42
41	5,2	15,7	43,9	8,8	281	283	50	1,38	33	42
42	4,7	13,7	41,6	7,4	250	478	32	1,15	0	41
43	4,4	13,6	40,4	9,7	310	313	33	1,36	30	214
44	4,8	15,1	44,1	10,2	275	189	21	0,97	11	45
45	4,8	14,0	40,8	7,7	198	181	44	1,08	9	43
46	4,0	14,0	41,1	5,8	214	79	44	1,08	0	50
47	4,6	14,9	44,2	15,3	197	333	49	1,66	33	49
48	5,0	15,1	45,2	10,4	354	181	40	1,40	0	57
49	4,5	13,2	38,9	5,0	221	234	30	1,00	23	23
50	5,2	14,1	41,7	5,0	204	129	36	0,98	28	25
51	4,4	14,2	41,4	7,1	171	73	36	1,56	0	44
52	4,7	14,8	43,0	6,4	277	225	43	1,27	7	37
53	4,5	14,2	41,3	15,4	365	366	29	1,33	0	42
54	4,8	14,2	42,0	7,2	224	226	33	1,03	0	32

ECC, erythrocyte count; HB, hemoglobin; HKT, hematocrit; WCC, white-cell count; PCC, platelet count; CK, creatine kinase; CR, creatinine; cTnI, cardiac troponin I; BNP, B-type natriuretic peptide.

Blood parameters (post-match)

n	ECC [million/mm^3]	HB [g/dl]	HKT [%]	WCC [thousand/mm^3]	PC [thousand/ mm^3]	CK [U/L]	Urea [U/L]	CR [U/L]	cTnI [ng/L]	BNP [pg/ml]
55	4,7	14,2	43,2	9,4	306	81	35	1,65	0	23
56	4,9	15,3	45,0	7,5	259	623	46	1,27	15	21
57	5,0	15,5	40,3	6,7	392	78	42	1,36	0	89
58	4,7	14,4	41,6	7,4	266	299	51	1,11	0	17
59	4,6	14,8	43,3	6,3	223	46	43	0,99	0	34
60	4,6	15,3	44,0	9,3	380	246	58	1,51	18	22
61	4,6	13,8	40,2	7,3	307	305	30	0,95	12	41
62	4,2	14,3	41,8	9,4	264	131	40	0,98	33	25
63	5,1	15,3	45,7	6,8	243	410	42	1,11	0	21
64	4,8	15,0	43,9	7,5	300	157	44	0,93	0	80
65	4,5	14,1	41,8	6,0	284	284	41	1,08	0	78
66	4,9	14,4	41,9	5,7	223	122	34	1,02	0	19
67	4,4	13,4	39,9	9,1	284	157	35	1,02	2	24
68	4,8	15,4	44,8	7,3	264	138	35	1,16	0	15
69	4,4	13,5	41,1	6,7	267	219	31	1,03	0	18
70	4,9	15,5	45,3	10,3	266	156	31	1,16	8	30
71	4,4	13,3	40,3	7,6	285	1148	30	1,13	0	31
72	4,3	13,6	41,0	7,0	274	326	41	1,25	5	32
73	5,2	16,0	46,1	13,3	318	252	40	1,22	8	22
74	4,9	15,0	44,2	7,3	230	190	46	1,35	10	14
75	4,6	14,4	43,5	5,8	259	234	36	1,08	0	27
76	5,2	15,8	46,0	5,8	193	184	45	1,23	0	19
77	4,7	14,5	42,0	5,1	251	73	40	1,17	0	42
78	4,4	13,9	41,5	11,4	332	176	39	1,23	40	21
79	4,8	14,4	42,7	7,7	267	111	45	0,96	0	53
80	4,9	15,1	44,4	12,0	216	569	28	1,25	0	23
81	4,8	15,2	45,0	7,4	256	464	36	1,30	0	28

ECC, erythrocyte count; HB, hemoglobin; HKT, hematocrit; WCC, white-cell count; PCC, platelet count; CK, creatine kinase; CR, creatinine; cTnI, cardiac troponin I; BNP, B-type natriuretic peptide.

Blood parameters (post-match)

n	ECC [million/mm^3]	HB [g/dl]	HKT [%]	WCC [thousand/mm^3]	PC [thousand/ mm^3]	CK [U/L]	Urea [U/L]	CR [U/L]	cTnI [ng/L]	BNP [pg/ml]
82	5,2	15,2	45,3	8,5	238	157	46	1,48	0	40
83	4,2	14,3	42,3	7,4	335	54	42	0,72	20	108
84	4,9	14,8	43,1	7,0	230	177	41	0,90	7	26
85	4,7	14,6	43,4	7,9	226	146	33	1,08	0	15
86	5,0	15,7	44,9	6,6	269	242	44	1,32	2	64
87	4,8	13,2	39,7	8,1	239	745	39	1,27	6	39
88	4,4	13,1	39,2	7,4	246	132	49	1,22	0	21
89	4,9	14,1	41,8	6,3	246	342	43	1,38	8	22
90	4,8	15,0	43,7	6,8	273	431	46	1,30	0	29
91	4,7	15,0	43,3	9,9	277	139	41	1,23	0	32
92	4,5	14,2	41,4	10,1	294	569	51	1,08	11	25
93	4,9	15,0	43,8	8,1	255	181	33	1,17	0	22
94	4,4	14,2	41,6	8,3	301	182	43	0,95	0	29
95	4,4	13,7	40,6	8,3	267	209	40	1,02	0	27
96	4,1	12,4	37,1	7,2	249	140	39	1,25	0	20
97	4,7	14,6	42,6	7,2	177	119	40	1,03	1	31
98	4,8	14,8	42,9	7,4	273	128	30	1,08	18	40
99	5,1	15,6	46,0	7,1	242	131	43	1,12	0	87
100	5,0	15,7	46,6	8,2	220	712	38	1,09	0	37
101	4,7	14,8	43,7	9,6	172	263	37	1,10	0	43
102	4,7	14,9	44,4	8,8	240	455	34	1,40	185	33
103	5,1	15,4	44,6	9,9	236	232	27	1,01	0	25
104	5,2	15,8	47,1	7,1	245	121	40	0,90	0	95
105	4,5	14,4	41,9	8,0	200	137	24	1,11	0	48
106	5,3	15,1	44,5	6,7	118	291	37	1,33	0	20
107	4,6	14,1	42,1	5,9	294	149	48	1,26	0	30
108	5,1	14,9	43,8	6,5	232	105	41	0,80	0	19

ECC, erythrocyte count; HB, hemoglobin; HKT, hematocrit; WCC, white-cell count; PCC, platelet count; CK, creatine kinase; CR, creatinine; cTnI, cardiac troponin I; BNP, B-type natriuretic peptide.

Blood parameters (post-match)

n	ECC [million/mm^3]	HB [g/dl]	HKT [%]	WCC [thousand/mm^3]	PC [thousand/ mm^3]	CK [U/L]	Urea [U/L]	CR [U/L]	cTnI [ng/L]	BNP [pg/ml]
109	4,83	15,0	43,9	6,6	308	634	40	1,51	0	19
110	4,99	15,3	45,4	5,6	203	124	35	0,97	0	63
111	5,10	15,3	45,0	7,4	294	149	59	1,64	0	27
112	4,62	14,7	43,8	6,7	326	187	36	1,00	0	42

ECC, erythrocyte count; HB, hemoglobin; HKT, hematocrit; WCC, white-cell count; PCC, platelet count; CK, creatine kinase; CR, creatinine; cTnI, cardiac troponin I; BNP, B-type natriuretic peptide.

cTnI-positive players (echocardiography)

n	AO [mm]	LA [mm]	IVSd [mm]	LVEDD [mm]	LVPWd [mm]	LVESD [mm]	EDV [ml]	ESV [ml]	LVEF [%]	FS [%]	RVEDD [mm]	E [cm/s]	A [cm/s]	E/A
1	29	43	11	57	11	38	160	61	62	34	19	8	9	0,9
2	25	37	13	39	12	24	68	19	72	40	25	9	10	0,9
3	29	37	10	51	10	29	124	31	75	44	28	15	10	1,5
4	32	36	9	54	10	27	143	27	81	50	15	11	11	1,0
5	36	34	11	47	10	28	100	29	71	40	25	8	11	0,7
6	26	29	9	50	8	28	116	30	74	43	22	14	11	1,2
7	37	41	10	53	10	34	138	46	67	37	25	11	8	1,5
8	33	47	10	53	10	34	137	46	66	37	30	10	7	1,3
9	34	39	10	52	9	30	130	34	74	43	25	11	10	1,1
10	35	37	10	57	8	38	162	61	62	34	24	11	11	1,0
11	30	35	10	49	10	31	112	39	65	36	18	12	14	0,8
12	31	37	9	55	8	38	148	61	59	31	27	17	11	1,5
13	32	37	10	48	9	29	108	33	70	30	26	7	10	0.7
14	31	35	11	48	11	27	108	27	75	44	24	13	11	1,2
15	33	39	10	56	9	35	154	51	67	38	24	10	9	1,2
16	36	35	12	53	11	41	156	76	59	22	19	6	12	0,5
17	32	37	11	54	10	32	142	42	71	41	25	11	11	1,0
18	32	37	10	52	10	32	129	41	68	38	25	11	11	1,0
19	39	37	10	54	10	32	139	42	70	40	32	12	7	1,7
20	33	37	10	58	9	33	165	45	73	42	26	9	11	0,8
21	32	37	10	41	10	25	72	22	70	39	20	9	14	0,6
22	28	31	9	47	8	25	102	22	78	47	25	11	12	0,9
23	33	32	9	52	10	37	131	59	55	29	25	12	13	0,9
24	34	37	11	51	10	30	123	34	73	42	30	12	13	1,0
25	32	36	10	59	10	41	174	72	59	31	26	5	10	0,6
26	28	43	10	56	10	33	153	44	71	41	20	14	10	1,5
27	36	39	10	52	10	32	129	41	68	38	25	10	14	0,7

AO, aortic diameter; LA, left atrial diameter; IVSd; septum thickness; LVEDD, left ventricular end-diastolic diameter; LVPWd, posterior wall thickness; LVESD, left ventricular end-systolic diamter; EDV, end-diastolic volume; ESV, end-systolic volume; LVEF, ejection fraction; FS, fractional shortening, E/A, transmitral peak early (E) to late (A) diastolic filling velocity.

cTnI-positive players (echocardiography)

n	AO [mm]	LA [mm]	IVSd [mm]	LVEDD [mm]	LVPWd [mm]	LVESD [mm]	EDV [ml]	ESV [ml]	LVEF [%]	FS [%]	RVEDD [mm]	E [cm/s]	A [cm/s]	E/A
28	29	43	11	57	11	38	160	61	62	34	19	8	9	0,9
29	25	37	13	39	12	24	68	19	72	40	25	9	10	0,9
30	29	37	10	51	10	29	124	31	75	44	28	15	10	1,5
31	32	36	9	54	10	27	143	27	81	50	15	11	11	1,0

AO, aortic diameter; LA, left atrial diameter; IVSd; septum thickness; LVEDD, left ventricular end-diastolic diameter; LVPWd, posterior wall thickness; LVESD, left ventricular end-systolic diamter; EDV, end-diastolic volume; ESV, end-systolic volume; LVEF, ejection fraction; FS, fractional shortening, E/A, transmitral peak early (E) to late (A) diastolic filling velocity.

cTnI-negative players (echocardiography)

n	AO [mm]	LA [mm]	IVSd [mm]	LVEDD [mm]	LVPWd [mm]	LVESD [mm]	EDV [ml]	ESV [ml]	LVEF [%]	FS [%]	RVEDD [mm]	E [cm/s]	A [cm/s]	E/A
1	27	32	18	37	10	27	111	52	53	27	28	8	12	0,7
2	31	33	9	51	9	31	121	38	68	38	30	10	11	0,8
3	35	40	11	56	11	32	52	42	72	42	27	12	9	1,3
4	37	33	9	50	9	34	121	46	61	33	28	9	8	1,2
5	33	40	10	52	9	33	127	43	66	36	27	6	11	0,6
6	32	40	10	44	12	29	87	32	63	34	30	10	11	0,8
7	31	41	9	54	9	37	142	58	59	32	26	10	10	1,1
8	29	0	9	49	9	33	112	45	60	32	28	15	15	1,0
9	33	36	10	52	10	33	127	43	66	36	26	10	11	0,9
10	39	35	10	55	9	37	145	57	61	33	27	12	10	1,2
11	31	41	10	58	12	35	163	50	69	40	26	10	10	0,9
12	34	37	10	50	8	28	121	30	75	44	29	8	13	0,6
13	36	43	11	62	11	39	191	66	65	36	24	10	9	1,1
14	32	34	10	50	9	29	120	32	73	42	18	9	13	0,7
15	38	39	10	52	10	33	127	43	66	36	27	12	13	0,9
16	34	46	10	55	9	36	145	54	63	34	30	8	10	0,7
17	33	42	11	53	10	31	134	38	72	41	35	11	10	1,1
18	28	36	11	53	11	32	133	40	70	40	19	9	14	0,6
19	28	36	10	52	10	31	130	37	72	41	24	12	12	1,1
20	34	38	11	55	10	33	147	45	69	39	23	12	9	1,3
21	35	36	12	49	11	37	111	57	49	25	27	8	10	0,8
22	31	35	11	48	10	29	108	33	70	39	31	7	11	0,6
23	32	37	10	51	10	32	121	42	66	36	25	11	10	1,1
24	29	41	9	50	10	27	116	26	78	46	26	13	14	1,0
25	33	36	10	56	9	32	151	40	73	43	22	12	10	1,2
26	39	34	9	53	10	30	136	35	75	44	30	8	11	0,7
27	31	33	9	53	9	37	136	57	58	31	22	11	12	0,9

AO, aortic diameter; LA, left atrial diameter; IVSd; septum thickness; LVEDD, left ventricular end-diastolic diameter; LVPWd, posterior wall thickness; LVESD, left ventricular end-systolic diamter; EDV, end-diastolic volume; ESV, end-systolic volume; LVEF, ejection fraction; FS, fractional shortening, E/A, transmitral peak early (E) to late (A) diastolic filling velocity.

cTnI-negative players (echocardiography)

n	AO [mm]	LA [mm]	IVSd [mm]	LVEDD [mm]	LVPWd [mm]	LVESD [mm]	EDV [ml]	ESV [ml]	LVEF [%]	FS [%]	RVEDD [mm]	E [cm/s]	A [cm/s]	E/A
28	32	36	12	53	12	24	136	20	0,85	0,55	24	10	16	0,6
29	33	35	11	54	12	33	143	43	0,70	0,40	37	9	10	0,9
30	31	41	9	50	8	31	120	37	0,69	0,39	27	13	11	1,2
31	36	40	11	60	9	37	182	59	0,68	0,38	28	7	16	0,5

AO, aortic diameter; LA, left atrial diameter; IVSd; septum thickness; LVEDD, left ventricular end-diastolic diameter; LVPWd, posterior wall thickness; LVESD, left ventricular end-systolic diamter; EDV, end-diastolic volume; ESV, end-systolic volume; LVEF, ejection fraction; FS, fractional shortening, E/A, transmitral peak early (E) to late (A) diastolic filling velocity.

cTnI-positive players (exercise stress test)

n	HR [bpm]	RR [mmHg]	QRS°	QRS [ms]	PQ [ms]	QTc [ms]	PVC [n]	HRmax [bpm]	RRmax [mmHg]	Pmax [W]	Pmax [W/kg]	cTnI pre [ng/L]	cTnI post [ng/L]	BNP pre [pg/ml]	BNP post [pg/ml]
1	54	140/90	39	94	157	411	1	162	230/90	283	3,1	7	36	54	53
2	82	160/100	45	91	157	409	1	174	220/100	268	2,0	4	15	32	40
3	66	110/80	41	99	160	390	0	187	200/90	200	2,2	2	0	22	29
4	62	120/80	43	99	170	410	0	192	240/90	258	3,3	0	13	38	28
5	54	130/80	-7	98	198	413	0	160	200/80	287	2,2	4	15	34	79
6	78	110/80	44	108	142	434	0	180	210/80	208	2,6	1	17	15	38
7	56	130/80	98	102	120	403	0	165	230/100	208	2,0	0	6	45	45
8	57	130/80	72	106	176	409	0	166	220/90	250	3,1	43	54	37	31
9	62	130/80	37	93	140	430	0	185	230/80	267	3,1	27	31	37	26
10	63	130/80	39	99	165	400	1	171	180/90	160	1,7	6	0	16	52
11	63	110/70	31	89	144	426	0	150	160/80	167	2,3	4	33	69	33
12	57	140/80	29	101	138	390	0	160	180/90	166	2,0	0	8	24	21
13	61	145/95	25	96	154	432	0	189	210/90	191	2,5	1	5	21	17
14	96	130/90	39	99	165	380	1	169	210/90	175	2,1	21	6	20	19
15	52	110/80	6	99	163	416	0	162	190/80	250	2,5	0	33	30	32
16	82	150/100	10	98	142	449	0	164	210/100	217	2,7	9	38	62	25
17	54	120/80	43	99	155	420	0	167	210/90	208	2,9	0	24	17	16
18	68	130/80	77	105	213	441	1	128	185/80	133	1,7	10	48	60	65
19	57	130/80	41	99	175	400	0	179	180/90	275	3,9	0	2	18	42
20	54	130/80	30	108	162	23	0	146	210/90	200	1,8	5	42	58	53
21	72	140/80	39	100	180	380	0	170	230/80	150	2,1	80	89	510	492
22	78	120/80	43	99	175	370	0	185	160/80	158	2,4	15	34	38	60
23	64	120/80	70	88	162	417	0	168	180/100	183	2,7	27	26	33	48
24	87	130/100	41	99	155	430	1	172	200/80	283	3,6	0	0	62	61
25	48	140/80	38	105	177	442	0	148	190/90	183	2,2	5	37	63	46
26	57	138/80	36	99	160	380	0	184	200/80	210	1,8	1	7	24	16
27	52	170/90	23	101	231	424	0	125	210/90	158	2,2	7	27	60	35

HR, heart rate at rest; RR, blood pressure at rest; QRS°, ECG axis; QRS/PQ/QTc time; PVC, premature ventricular contraction; HRmax, maximum heart rate; RRmax, maximum blood pressure; Pmax, maximum performance; cTnI (cardiac troponin I) and BNP (B-type natriuretic peptide) pre and post exercise stress test.

cTnI-positive players (exercise stress test)

n	HR [bpm]	RR [mmHg]	QRS°	QRS [ms]	PQ [ms]	QTc [ms]	PVC [n]	HRmax [bpm]	RRmax [mmHg]	Pmax [W]	Pmax [W/kg]	cTnI pre [ng/L]	cTnI post [ng/L]	BNP pre [pg/ml]	BNP post [pg/ml]
28	61	170/100	33	95	174	452	0	150	280/100	200	2,6	6	45	56	45
29	72	100/65	48	89	149	398	0	168	160/90	217	2,5	35	42	41	29
30	55	130/80	41	99	165	400	0	170	280/100	233	2,8	1	28	24	19
31	54	150/110	86	108	191	428	0	147	210/110	161	2,1	0	0	20	21

HR, heart rate at rest; RR, blood pressure at rest; QRS°, ECG axis; QRS/PQ/QTc time; PVC, premature ventricular contraction; HRmax, maximum heart rate; RRmax, maximum blood pressure; Pmax, maximum performance; cTnI (cardiac troponin I) and BNP (B-type natriuretic peptide) pre and post exercise stress test.

cTnI-negative players (exercise stress test)

n	HR [bpm]	RR [mmHg]	QRS°	QRS [ms]	PQ [ms]	QTc [ms]	PVC [n]	HRmax [bpm]	RRmax [mmHg]	Pmax [W]	Pmax [W/kg]	cTnI pre [ng/L]	cTnI post [ng/L]	BNP pre [pg/ml]	BNP post [pg/ml]
1	75	110/70	-27	173	172	449	0	157	110/70	150	2,0	62	9	16	12
2	63	130/80	3	102	151	439	0	178	130/80	225	3,0	34	0	21	20
3	50	120/85	31	110	162	386	0	192	120/85	275	2,9	55	5	38	27
4	58	110/80	45	86	137	381	0	174	110/80	200	2,8	12	31	59	50
5	56	130/80	35	103	137	385	1	161	130/80	233	3,2	24	8	27	29
6	62	125/80	27	118	145	411	0	166	125/80	200	2,6	4	30	35	35
7	66	120/80	44	102	155	417	0	180	120/80	266	3,2	38	11	14	16
8	62	130/85	34	91	146	414	0	162	130/85	183	2,6	0	0	42	24
9	55	120/70	5	102	225	403	0	166	120/70	216	3,3	0	0	21	20
10	61	120/80	43	101	158	399	0	172	120/80	233	3,1	0	0	21	12
11	57	140/80	12	100	152	438	1	160	140/80	283	3,0	18	0	53	62
12	53	110/80	-19	109	183	398	0	171	110/80	200	2,4	16	3	23	23
13	65	150/90	13	102	160	485	0	155	150/90	233	2,8	0	0	56	41
14	87	130/75	-2	100	153	442	1	172	130/75	200	2,5	0	0	16	15
15	61	120/80	50	95	190	417	0	163	120/80	283	3,5	0	0	98	84
16	56	135/80	-11	97	183	425	0	139	135/80	250	2,3	0	0	70	45
17	57	130/90	-1	94	140	401	0	145	130/90	221	2,2	0	0	35	23
18	62	165/90	-7	94	161	424	0	154	165/90	208	2,4	0	0	24	30
19	54	140/90	45	95	131	423	0	172	140/90	250	2,6	0	0	16	22
20	49	130/90	71	118	150	465	0	167	130/90	233	2,8	7	4	50	26
21	59	145/100	-30	90	169	408	0	168	145/100	225	2,6	0	27	25	23
22	79	120/80	38	109	143	425	0	178	120/80	183	2,6	13	0	15	17
23	65	140/90	51	102	145	400	0	170	140/90	250	2,7	7	0	37	13
24	69	130/90	45	105	139	413	1	170	130/90	158	2,6	77	37	88	20
25	59	130/80	39	91	160	387	0	198	130/80	200	2,9	1	0	27	23
26	74	145/80	48	94	178	423	0	158	145/80	200	2,5	0	0	21	19
27	72	160/100	-10	99	135	438	0	171	160/100	216	3,1	0	0	12	10

HR, heart rate at rest; RR, blood pressure at rest; QRS°, ECG axis; QRS/PQ/QTc time; PVC, premature ventricular contraction; HRmax, maximum heart rate; RRmax, maximum blood pressure; Pmax, maximum performance; cTnI (cardiac troponin I) and BNP (B-type natriuretic peptide) pre and post exercise stress test.

cTnI-negative players (exercise stress test)

n	HR [bpm]	RR [mmHg]	QRS°	QRS [ms]	PQ [ms]	QTc [ms]	PVC [n]	HRmax [bpm]	RRmax [mmHg]	Pmax [W]	Pmax [W/kg]	cTnI pre [ng/L]	cTnI post [ng/L]	BNP pre [pg/ml]	BNP post [pg/ml]
28	89	160/95	68	89	199	392	0	145	280/90	150	1,6	0	3	20	17
29	62	120/70	-16	107	127	462	1	176	180/80	250	2,8	0	0	20	22
30	58	120/80	67	105	186	417	0	167	210/80	208	2,5	0	0	42	37
31	75	150/85	32	111	149	437	1	158	130/80	175	2,0	35	9	56	48

HR, heart rate at rest; RR, blood pressure at rest; QRS°, ECG axis; QRS/PQ/QTc time; PVC, premature ventricular contraction; HRmax, maximum heart rate; RRmax, maximum blood pressure; Pmax, maximum performance; cTnI (cardiac troponin I) and BNP (B-type natriuretic peptide) pre and post exercise stress test.
